# Cationic nanoparticles enhance T cell tumor infiltration and antitumor immune responses to a melanoma vaccine

**DOI:** 10.1126/sciadv.abk3150

**Published:** 2022-07-20

**Authors:** Rasheid Smith, Emad I. Wafa, Sean M. Geary, Kareem Ebeid, Suhaila O. Alhaj-Suliman, Aliasger K. Salem

**Affiliations:** Department of Pharmaceutical Sciences and Experimental Therapeutics, College of Pharmacy, University of Iowa, Iowa City, IA 52242, USA.

## Abstract

In clinical settings, cancer vaccines as monotherapies have displayed limited success compared to other cancer immunotherapeutic treatments. Nanoscale formulations have the ability to increase the efficacy of cancer vaccines by combatting the immunosuppressive nature of the tumor microenvironment. Here, we have synthesized a previously unexplored cationic polymeric nanoparticle formulation using polyamidoamine dendrimers and poly(d,l-lactic-*co*-glycolic acid) that demonstrate adjuvant properties in vivo. Tumor-challenged mice vaccinated with an adenovirus-based cancer vaccine [encoding tumor-associated antigen (TAA)] and subsequently treated with this nanoparticulate formulation showed significant increases in TAA-specific T cells in the peripheral blood, reduced tumor burden, protection against tumor rechallenge, and a significant increase in median survival. An investigation into cell-based pathways suggests that administration of the nanoformulation at the site of the developing tumor may have created an inflammatory environment that attracted activated TAA-specific CD8^+^ T cells to the vicinity of the tumor, thus enhancing the efficacy of the vaccine.

## Teaser

Cationic nanoparticles increase adenovirus cancer vaccine efficacy.

## INTRODUCTION

Cancer immunotherapy represents an important treatment strategy for patients with inoperable cancers such as late-stage melanoma (*1*). A primary goal of cancer immunotherapy is to break immune tolerance in the immunosuppressive tumor microenvironment (TME) and mount an efficient immune effector response against tumor cells (*2*). Several U.S. Food and Drug Administration (FDA)–approved cancer immunotherapy strategies exist for melanoma treatment including checkpoint blockade [ipilimumab (anti–CTLA-4) and pembrolizumab (anti–PD-1)], oncolytic viral therapy (talimogene laherparepvec), and combinational checkpoint blockade [nivolumab (anti–PD-1) + ipilimumab] (*3*). One approach that has shown great promise in preclinical therapeutic settings is the use of cancer vaccines where adjuvants in combination with tumor-specific antigens (TSAs) or tumor-associated antigens (TAAs) are administered to generate tumor-specific cytotoxic T cell responses (*2*, *4*). Currently, Sipuleucel-T is the only FDA-approved therapeutic cancer vaccine; this involves the reinfusion of the host’s blood cells [containing dendritic cells (DCs)] pretreated and activated with a recombinant fusion antigen [granulocyte-monocyte colony-stimulating factor (GM-CSF)–prostatic acid phosphatase]. However, its marginal increase in median survival time (4.1 months) (*5*) and the high cost (≈$100,000 per patient) prohibit its widespread adoption. Currently, a personalized DC-based melanoma cancer vaccine is in a phase 2b clinical trial (NCT02301611) but runs the risk of following the path of Sipuleucel-T by having a high cost associated with it, thus making it inaccessible to most patients not adequately insured.

A viable and cost-effective alternative to cell-based vaccines that can be delivered without the need for harvesting tissues from the patient includes using viral-based vaccines; these are an efficient means of delivering DNA encoding TAAs or TSAs and eliciting effective cytotoxic T lymphocyte (CTL) responses (*6*). While each viral vector has its advantages and disadvantages, the replication-deficient adenovirus has been proven to have many advantages, and its few disadvantages can be readily surmounted. The replication-deficient serotype 5 adenovirus (Ad5) has well-documented production techniques, can produce high viral titer stocks, and can encode relatively large DNA inserts, which can result in multiple whole-TAA/TSA expression (*7*). Along with high-efficiency gene transduction, the Ad5 has been shown to have a tropism for DCs, the most potent professional antigen-presenting cell (APC) population (*8*, *9*). Given that DCs are the basis for all current cell-based cancer vaccines with FDA approval for those in clinical trials, the Ad5 cancer vaccine may be a viable and less expensive alternative (*5*, *7*, *10*, *11*). Ad5 has also been proven to be well tolerated while being highly immunogenic in humans (*12*, *13*). An unfortunate downside to using Ad5 is the reduction in efficacy due to neutralizing antibodies resulting from prior exposure to the wild-type virus; however, this can be readily circumvented using a gelatin matrix such as Gelfoam to deliver the Ad5-based vaccine (*14*–*16*).

Ideally, TSAs would be preferred over TAAs as the immunogen of choice for a cancer vaccine because of their inherently greater immunogenicity (*2*, *17*, *18*). However, TSAs lack the possibility of being prepared on a large standardized scale as they are often patient specific (*18*). Also, TAA-based vaccines can be produced ahead of diagnosis since it is already known that a patient diagnosed with a certain type of cancer will likely express well-defined TAAs. For example, patients with melanoma will have tumors expressing a suite of defined TAAs [including tyrosinase-related protein 2 (TRP2)]. However, regardless of whether TAA-based or TSA-based cancer vaccines are implemented, they both must overcome the myriad of immunosuppressive properties of the TME (*19*). Numerous strategies have been tested in preclinical studies in an attempt to improve the potency of TAA-based and model TSA-based cancer vaccines (*2*, *20*, *21*). It has been previously demonstrated that combining the Ad5 cancer vaccine (encoding a model TSA) with intratumoral administration of the adjuvant cytosine guanine oligonucleotide (CpG ODN) significantly reduces tumor growth and increases survival in mice, along with increasing the proportion of antigen-specific CD8^+^ T cells in the TME and peripheral blood (*15*, *22*). Adjuvants formulated into nanoparticles (NPs) may be effective at modulating the immunosuppressive nature of the TME. Studies have demonstrated the increased efficacy of adjuvants when formulated into NPs (*4*, *23*), and NPs of less than 500 nm have been shown to effectively accumulate in DCs (*24*).

Thus, formulating an NP-based adjuvant system may provide a possible means to further enhance the immunogenicity of Ad5-based cancer vaccines and cancer vaccines in general. Nanoscopic compounds such as dendrimers have created new avenues toward the development of novel delivery systems (*25*). Since being introduced in 1984, polyamidoamine dendrimers (PAMAMs) have gained the attention of many researchers as a tool for gene delivery (*26*–*28*) and drug delivery (*29*–*31*). Despite their great potential and biomedical applications, PAMAMs are known to be toxic; however, this can be overcome through chemical modifications that shield the highly cationic surface (*32*, *33*). This, of course, limits the very attributes that differentiated PAMAM to begin with. In an effort to yield the beneficial attributes of PAMAM without reducing their biomedical application through chemical modification, we have developed a previously unexplored PAMAM-based NP formulation using a combination of poly(d,l-lactic-*co*-glycolic acid) (PLGA) and PAMAM to form NPs that limit the toxicities associated with unmodified PAMAM. This NP differs from previously reported formulations where PAMAM was physically adsorbed to the surface of PLGA NPs (*34*), or the PAMAM was chemically modified to yield beneficial attributes. This PLGA/PAMAM NP formulation will be termed PM and represents the first time that PLGA and PAMAM were used to make bilayer NPs from a single polymer solution that contains both polymers. To investigate the potential adjuvant properties of PM, we have used them in combination with an antitumor vaccine, Ad5 encoding the melanoma TAA, tyrosinase-related protein 2 (Ad5-TRP2). Melanoma represents an appropriate model to explore the effectiveness of this formulation for several reasons. The National Institutes of Health Surveillance and Epidemiology and End Results Program places malignant melanoma as the fifth most commonly diagnosed cancer in the United States, with an estimated 96,480 new cases in 2019 (*35*). Melanoma, if detected early (melanoma in situ), can be treated with surgery or targeted therapeutic agents based on a patient’s mutation status; however, for advanced-stage melanoma (stage III or IV), few options are available to patients (*36*). Thus, immunotherapy represents a viable alternative for these patients. Here, we have demonstrated that therapeutically combining PM with Ad5-TRP2 resulted in inhibition of tumor growth and increased survival in melanoma-challenged mice, which was, in part, due to greatly increased systemic CD8^+^ T cell responses and to the enhanced accumulation of CD8^+^ T cells to the tumor and tumor periphery. Mice that survived tumor-free (TF) long-term after receiving this treatment were also protected from tumor rechallenge.

## RESULTS

### Particle synthesis, characterization, and uptake

**Fig. 1.**
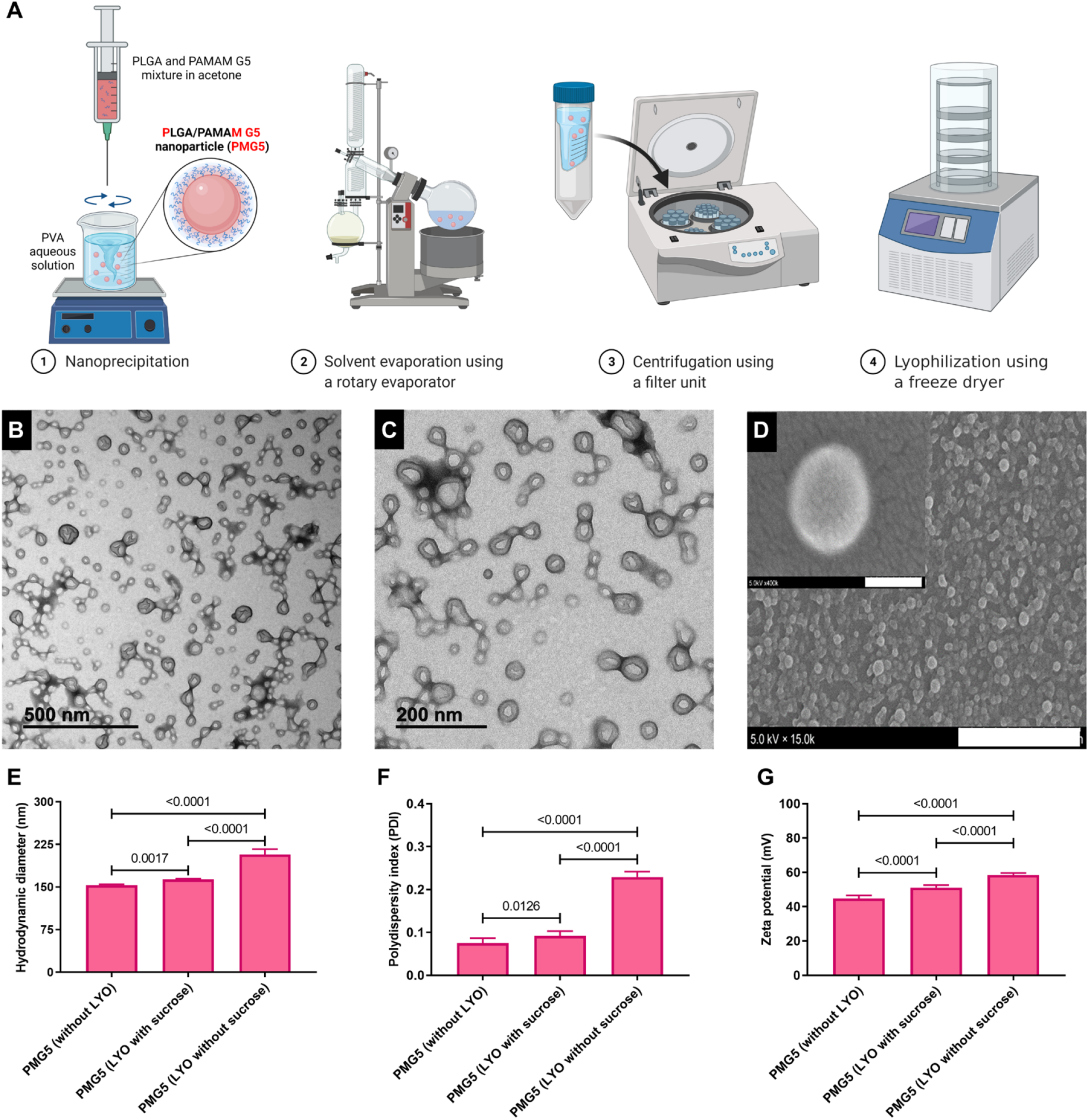
Preparation and characterization of PMG5. (**A**) Diagram summarizing the major stages involved in the synthesis of PMG5. (**B** and **C**) TEM images of PMG5 at different magnifications (negative stain, 1.5% phosphotungstic acid). (**D**) Scanning electron photomicrograph of PMG5. Scale bars, 3 μm and (inset) 100 nm. (**E**) Graph showing the hydrodynamic diameter of the indicated groups. (**F**) Graph showing the size distribution (PDI) of various groups. (**G**) Graph showing the net surface charge (zeta potential) of the indicated groups. Data are plotted as means ± SD. Error bars represent SD, *n* = 9. LYO, lyophilization.

**Fig. 2.**
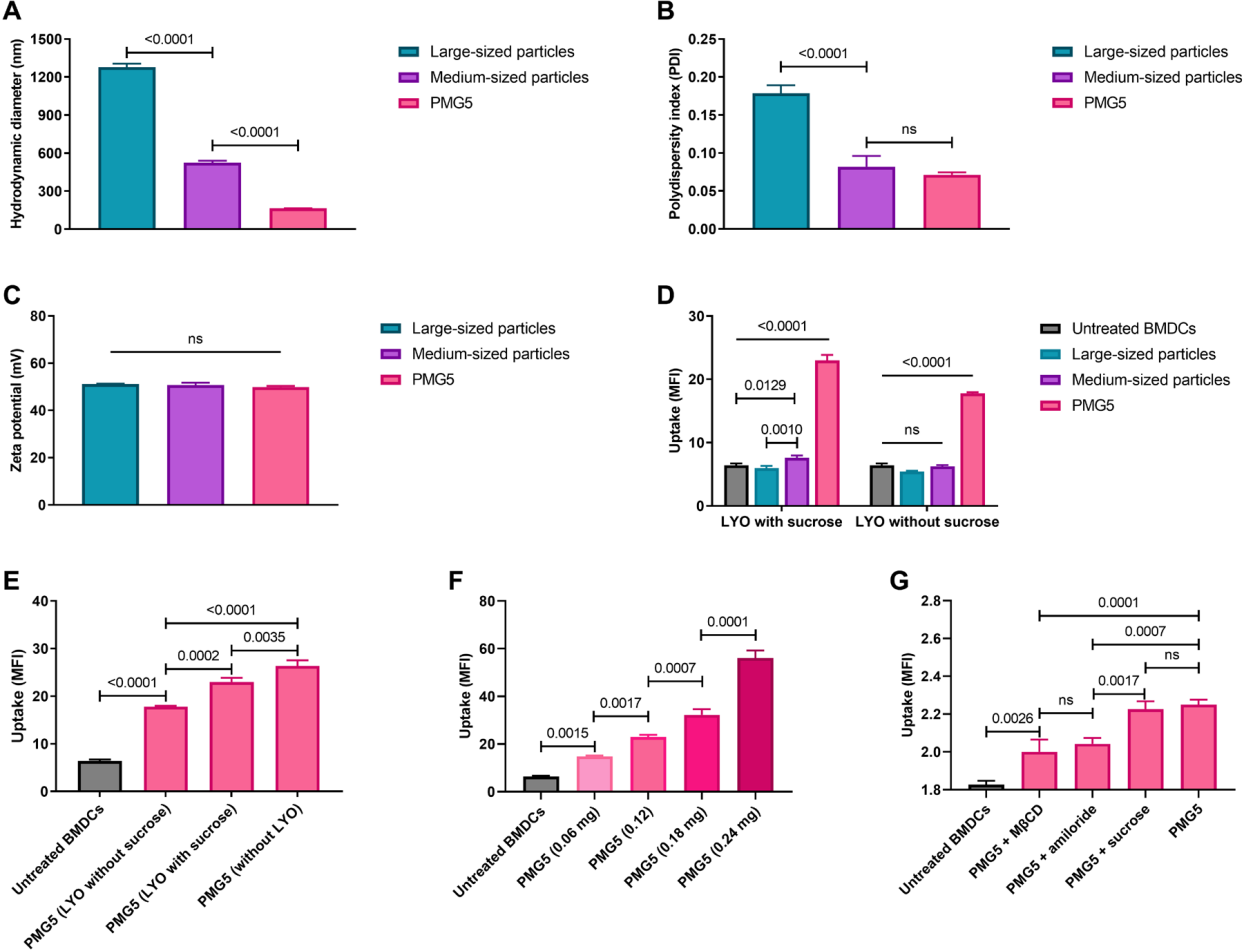
Evaluation of the three PLGA/PAMAM-based formulations with three different particle sizes. (**A**) Graph showing the size of the particles. (**B**) Graph showing the size distribution (PDI) of particles. (**C**) Graph showing the net surface charge (zeta potential) of particles. (**D**) Graph showing the uptake of the three formulations by BMDC after 48 hours of incubation in vitro. (**E**) Graph showing the effect of lyophilization conditions on the uptake of PMG5 by BMDCs after 48 hours of incubation in vitro. (**F**) Graph showing the dose-uptake relationship of PMG5 by BMDCs after 48 hours of incubation in vitro. (**G**) Graph showing the effects of various inhibitors on the uptake of PMG5 by BMDCs after 3 hours of incubation in vitro. Data are plotted as means ± SD. Error bars represent SD, *n* = 9. Probability was determined by one-way ANOVA with Tukey posttest. Differences in the scale of MFI between graphs in (F) and (G) are due to the differences in incubation times (48 versus 3 hours, respectively). Large- and medium-sized particles were micrometer-sized and submicrometer-sized particles, respectively, that were also made from PLGA and PAMAM in the same ratios described in Materials and Methods. ns, not significant.

PAMAM can be classified according to a generation number, referring to the degree of branching from the core and being related to the number of surface amines present on the PAMAM. PMs were synthesized using the nanoprecipitation method summarized in Fig. 1A from a mixture of PLGA and PAMAM (ethylenediamine core, generation 5), and the resultant NPs will be referred to as PMG5. Transmission electron microscopy (TEM) and scanning electron microscopy (SEM) images showed that PMG5 were spherical with smooth surfaces (Fig. 1, B to D). Fabricating PMG5 before each use is not practical for translation into the clinic. Thus, to evaluate their stability under appropriate storage conditions, PMG5 were lyophilized (freeze-dried) with and without sucrose, a cryoprotectant. The lyophilization of PMG5 without sucrose resulted in significant aggregation as indicated by an increase in the size and polydispersity index (PDI) of the PMG5 (Fig. 1, E and F). This effect was circumvented by adding sucrose to the formulation, which resulted in a significant decrease in the size and PDI of PMG5 when compared to PMG5 lyophilized without sucrose. It has been previously established that PAMAMs exhibit intrinsic fluorescence in the blue region with a slight shift in lambda max with increasing generations (*37*–*39*). Taking advantage of this feature, the intrinsic fluorescence of PAMAM was used to evaluate the uptake of PMG5 by bone marrow–derived DCs (BMDCs), and fluorescence (an indicator of uptake) was assessed by flow cytometry. Results shown in Fig. 2D demonstrate clearly that BMDCs incubated with PMG5 (163.9 ± 0.61 nm) had significantly greater fluorescence (due to the PMG5 being taken up by BMDCs) than BMDCs incubated with larger submicrometer-sized (523.9 ± 15 nm) and micrometer-sized particles (1278.3 ± 27 nm) (also made from PLGA and PAMAM) after 48 hours of incubation.

The effect of using sucrose as a stabilizer during lyophilization on PMG5 uptake by BMDCs was further evaluated by incubating BMDCs with freshly prepared PMG5 and PMG5 lyophilized with and without sucrose. It was observed that PMG5 lyophilized with sucrose were taken up more efficiently by BMDCs compared to PMG5 lyophilized without sucrose (Fig. 2E). Thus, it would appear that because sucrose preserved the size and PDI of lyophilized PMG5 (and therefore were similar to freshly prepared PMG5 as shown in Fig. 1), their capacity to be taken up by BMDCs was also mostly preserved (Fig. 2E). Thus, PMG5 lyophilized with sucrose were used in subsequent studies. Treatment of BMDCs with different doses of PMG5 revealed a dose-dependent rate of uptake (Fig. 2F). The uptake mechanism of PMG5 was investigated by incubating PMG5 with BMDCs pretreated with inhibitors of three distinct pathways of endocytosis: amiloride (an inhibitor of macropinocytosis), methyl-β-cyclodextrin (MβCD) (an inhibitor of clathrin-independent endocytosis), and a high concentration (400 μM) of sucrose (an inhibitor of clathrin-mediated endocytosis). As expected, incubation of BMDCs (not treated with any inhibitor) with PMG5 displayed a significant increase in the mean fluorescence intensity (MFI) compared to untreated BMDCs (cells not incubated with PMG5) (Fig. 2G). Incubation of PMG5 with BMDCs pretreated with a high concentration of sucrose did not result in a significant reduction in the MFI when compared to BMDCs incubated with PMG5 in the absence of an inhibitor. PMG5 incubated with BMDCs pretreated with either amiloride or MβCD exhibited a significant reduction in the MFI in comparison to PMG5 incubated with BMDCs (without inhibitors). This indicated that both clathrin-independent endocytosis and macropinocytosis likely contribute to the uptake of PMG5 by BMDCs.

### PMG5 thermal stability

A differential scanning calorimetry (DSC) thermogram of PLGA indicated that the polymer had a glass transition temperature of ~45°C (representing the amorphous region of PLGA) (fig. S1A). Adding the PAMAM G5 to PLGA (i.e., physical mixture) had no effect on the thermal properties of the PLGA. When PLGA was incorporated into NPs (with and without sucrose) using the nanoprecipitation technique, it did not exhibit any changes in thermal properties when compared to unprocessed PLGA, except for a slight shift in the glass transition temperature to ~49°C. The DSC thermogram demonstrated that the PMG5 formulation is thermally stable at the ambient temperature since it did not display any thermal events in the range of 0° to 49°C. It is likely that the highly positive surface charge of PMG5 helped to prevent aggregation in solution at room temperature due to repulsive forces (fig. S1B). At 37°C, PMG5 dispersed in water showed no significant change in size, PDI, and surface charge for at least 10 days of stirring at 300 rpm (fig. S1, B to D); however, after 10 days, the hydrodynamic size (and PDI) did increase, most likely due to aggregation.

### Antitumor efficacy of Ad5-TRP2 combined with PMG5

**Fig. 3.**
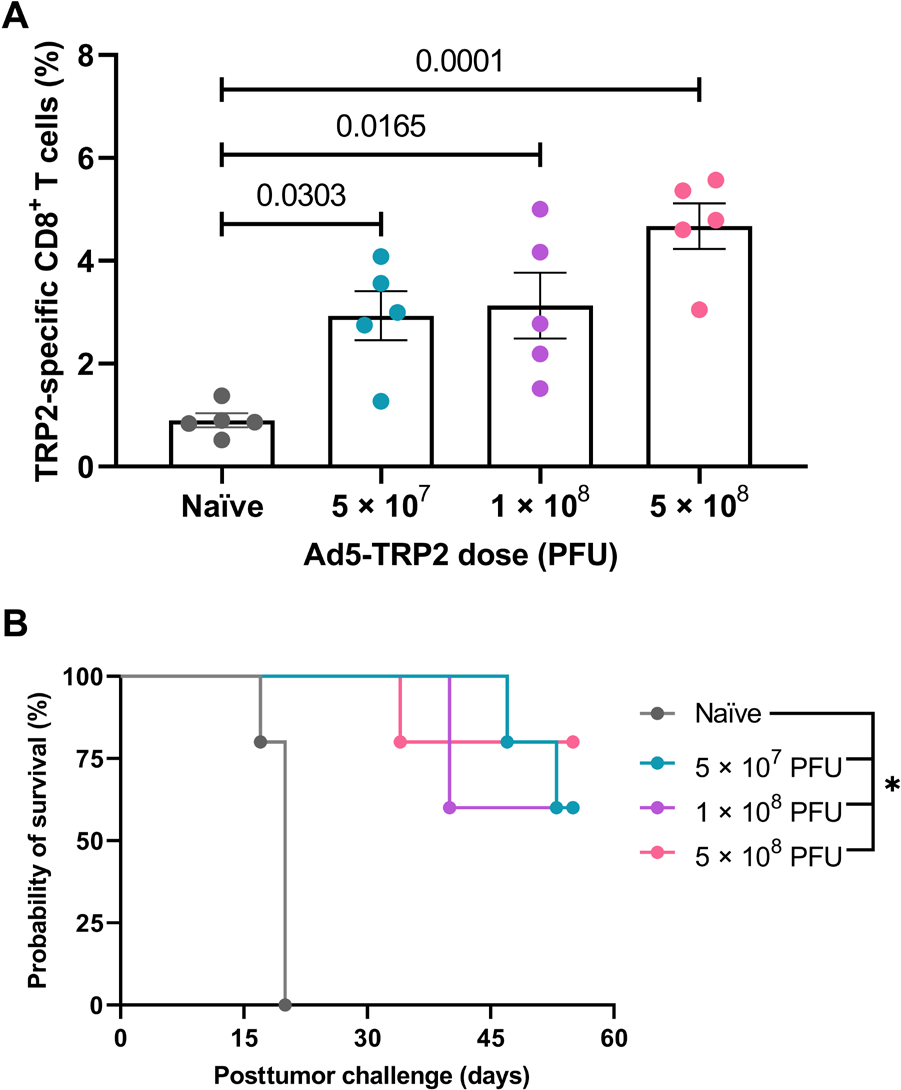
Prophylactic immunization of C57BL/6J mice with varying doses of Ad5-TRP2. Mice (*n* = 5 per group) were vaccinated subcutaneously with the indicated dose of Ad5-TRP2 and then (**A**) assessed for levels of TRP2-specific CD3^+^CD8^+^ T lymphocytes (on day 14 after vaccination) using direct immunostaining (see Materials and Methods) and flow cytometry (expressed as a percentage of total CD3^+^CD8^+^ T lymphocytes). Error bars represent SD. *P* values were determined by one-way ANOVA with Tukey posttest. On day 14 after vaccination, all mice were challenged with B16.F10 cells, and (**B**) survival was recorded and a survival graph generated using Prism software. *P < 0.05 (for all treated groups compared to the naïve group) as determined by the log-rank test adjusted for multiple comparisons.

Initially, prophylactic studies were performed and demonstrated that vaccinating nontumor-challenged mice with Ad5-TRP2 at 5 × 10^7^, 1 × 10^8^, or 5 × 10^8^ plaque-forming units (PFUs) led to significantly enhanced levels of TRP2-specific CD8^+^ T lymphocytes in the peripheral blood compared to unvaccinated mice on day 14 after vaccination (Fig. 3A). No significant differences were seen between the different doses, although a trend toward a dose-dependent response was recognized. However, unpaired *t* tests revealed a significant difference between 5 × 10^7^ and 5 × 10^8^ PFU vaccinations (*P* = 0.031) but no significance when 1 × 10^8^ and 5 × 10^8^ PFU vaccinations were compared (*P* = 0.098). These same mice were also challenged subcutaneously with live B16.F10 cells (1 × 10^5^ cells per mouse) on day 14 after vaccination, and all three vaccination doses showed a protective phenotype with all control mice (100%, *n* = 5) succumbing to the tumor by day 20 posttumor challenge (PTC), while the mice vaccinated with 5 × 10^7^, 1 × 10^8^, and 5 × 10^8^ PFUs of Ad5-TRP2 demonstrated 60, 60, and 80% TF survival at the termination of the experiment on day 55, respectively (Fig. 3B).

**Fig. 4.**
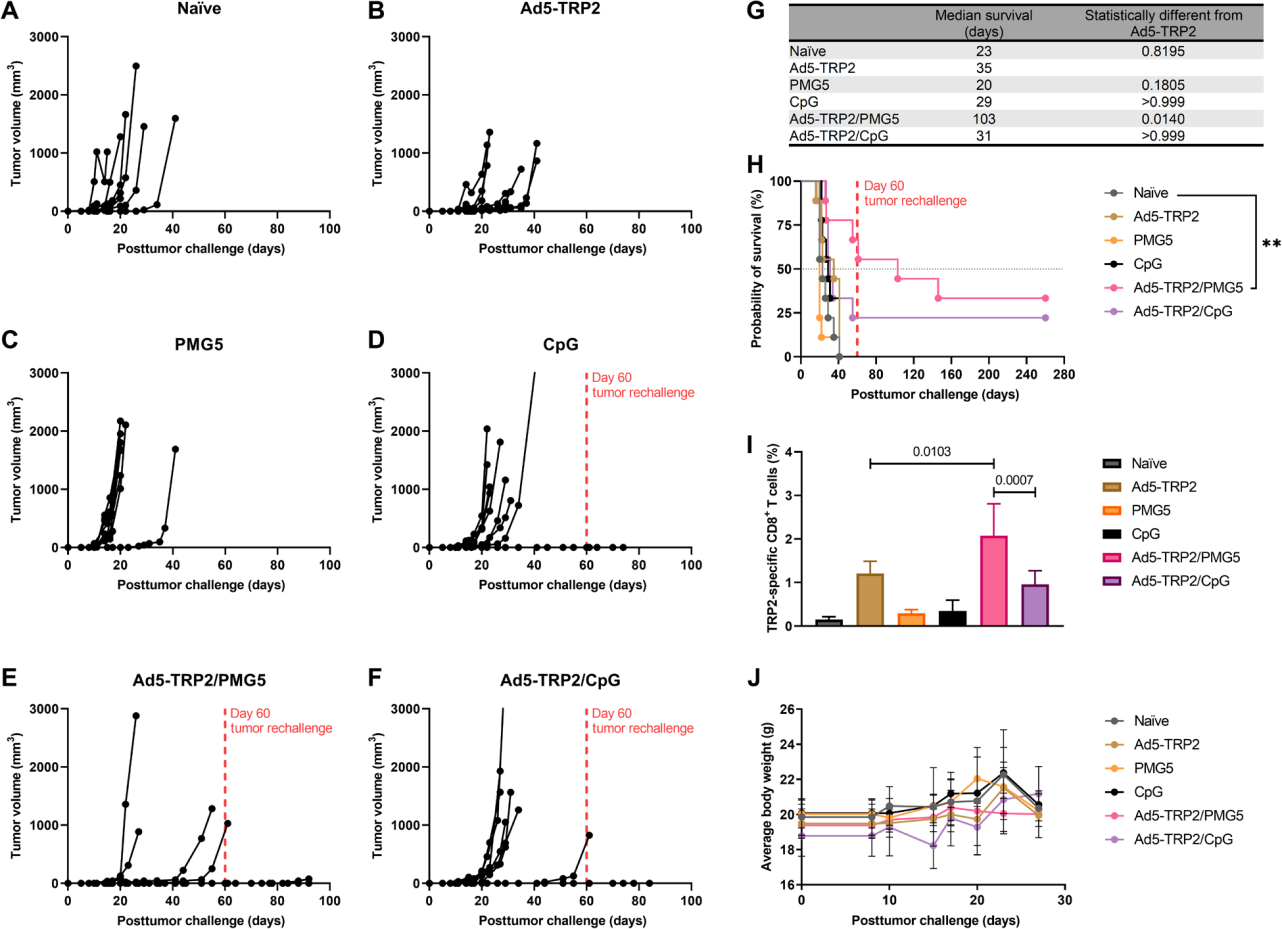
Therapeutic immunizations of C57BL/6J mice with Ad5-TRP2 and peritumoral administrations of PMG5. (**A** to **F**) Tumor volume curves of mice treated with the designated treatment. (**G**) Table showing the median survival of mice. (**H**) Survival curve of mice treated with different treatments after being challenged with tumors. **P < 0.01. (**I**) Graph showing the percent of TRP2-specific CD8^+^ T cells in the peripheral blood (14 days after tumor challenge). (**J**) Graph showing the average weight of mice over time. Data are plotted as means ± SD. For (H), the probability was determined by the log-rank test with all groups compared to the naïve group, adjusted for multiple comparisons. For (I), the probability was determined by one-way ANOVA with Tukey posttest.

In a therapeutic setting, there was a marginal but nonsignificant increase in TRP2-specific CD3^+^CD8^+^ T lymphocytes when the two doses of Ad5-TRP2 (1 × 10^8^ versus 5 × 10^8^ PFUs) were compared (fig. S2), as was also observed in the prophylactic setting (Fig. 3A). Thus, for subsequent therapeutic studies, mice were vaccinated with 1 × 10^8^ PFUs of Ad5-TRP2 unless otherwise stated. To evaluate the effect of PMG5 on Ad5-TRP2 vaccination therapeutically, mice were challenged subcutaneously with B16.F10 cells on day 0 and then, on day 1 PTC, vaccinated subcutaneously with 1 × 10^8^ PFUs of Ad5-TRP2 in the contralateral flank, followed by peritumoral administration of PMG5 on days 8, 11, and 13 PTC. Figure 4I demonstrates that mice in the Ad5-TRP2/PMG5 treatment group had a significant increase in the percent of TRP2-specific CD3^+^CD8^+^ T lymphocytes in the peripheral blood versus mice in the Ad5-TRP2 treatment group (2.08 ± 0.73 versus 1.21 ± 0.28%, respectively; *P* = 0.0176). In comparison, mice treated with Ad5-TRP2/CpG had only 0.96 ± 0.31% TRP2-specific CD3^+^CD8^+^ T lymphocytes in their peripheral blood (*P* = 0.0073: compared to the Ad5-TRP2/PMG5 treatment group). Mice treated with Ad5-TRP2/PMG5 also exhibited reduced tumor growth rates compared to naïve mice (Fig. 4, A versus E), with five of nine mice remaining TF for 90 days PTC (after a secondary tumor challenge at 60 days PTC). Mice treated with Ad5-TRP2/CpG had only two of nine mice remaining TF 90 days PTC. When compared to all other treatment groups, the Ad5-TRP2/PMG5 group provided the most protection against tumor growth. This ultimately translated into Ad5-TRP2/PMG5 mice being the only group with a significant increase in median survival of mice when compared to the Ad5-TRP2 mice (103 days for Ad5-TRP2/PMG5–treated mice versus 35 days for naïve mice; *P* = 0.0140) (Fig. 4G). To evaluate whether treatments given to mice generated immunological memory protecting mice from future tumor challenges, surviving mice that were TF at day 60 were rechallenged with B16.F10 cells (Fig. 4H). Ad5-TRP2/PMG5 TF mice (*n* = 5) did not develop tumors 30 days after tumor rechallenge. In addition, Ad5-TRP2/CpG TF mice (*n* = 2) also did not develop tumors within this time frame (Fig. 4H). The treatments demonstrated no signs of toxicity as determined by weight measurements and monitoring for signs of distress (Fig. 4J).

### DC activation

**Fig. 5.**
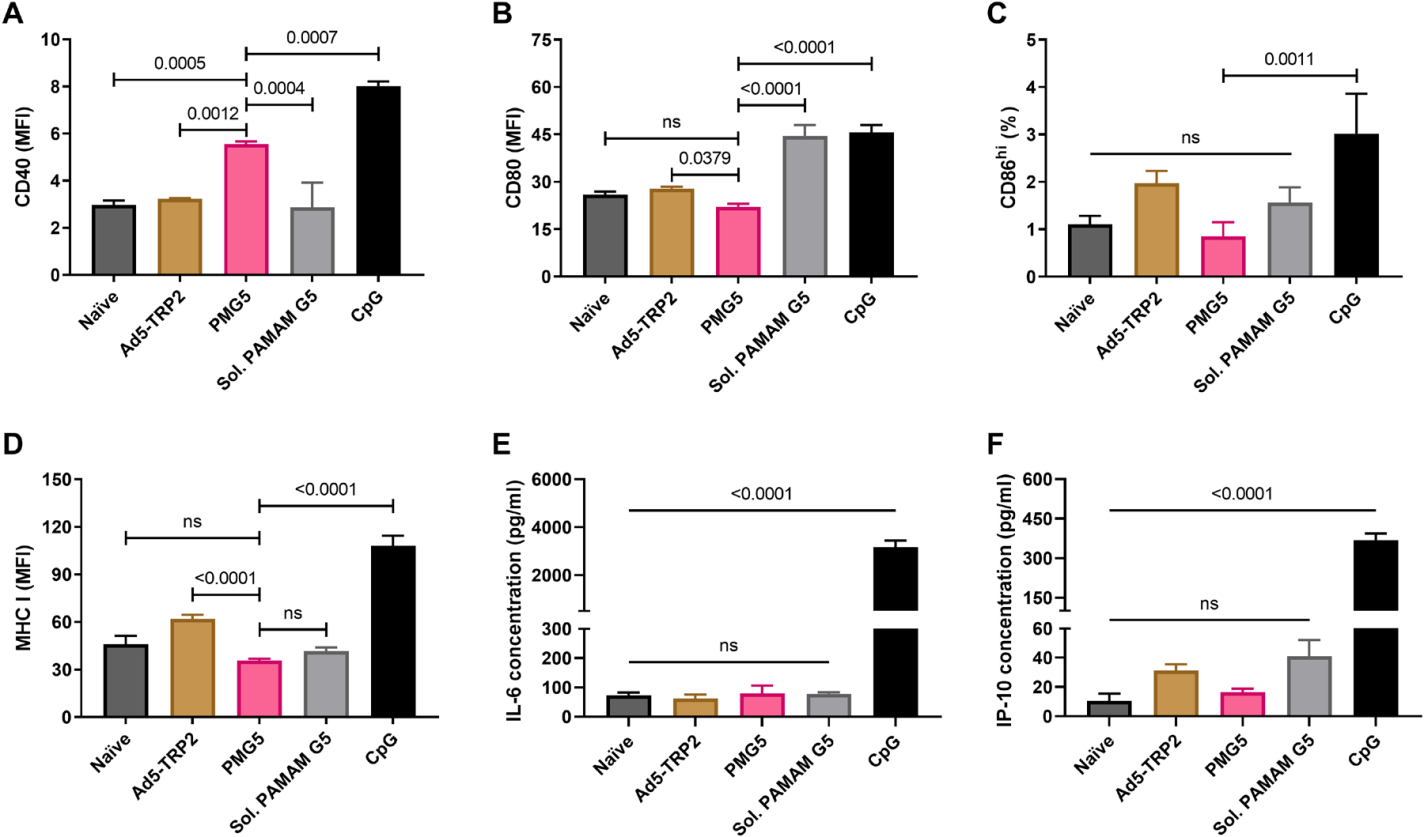
In vitro stimulation of BMDCs. BMDCs were incubated for 2 days with the indicated treatment (as described in Materials and Methods), and then cells and supernatants were harvested for analysis of surface marker expression and cytokine secretion, respectively. (**A**) Graph showing relative CD40 expression. (**B**) Graph showing relative CD80 expression. (**C**) Graph showing percent CD86^hi^ expression. (**D**) Graph showing relative MHC class I expression. (**E**) Graph showing IL-6 secretion levels. (**F**) Graph showing difference in IP-10 secretion. Data are plotted as means ± SD. Probability values are determined by one-way ANOVA with Tukey posttest.

Having established that BMDCs can take up PMG5 (Fig. 2D) and that PMG5 could enhance the therapeutic activity of Ad5-TRP2 (Fig. 4I), we wished to evaluate the impact that PMG5 has on the activation/maturation of BMDCs. Thus, BMDCs were incubated with PMG5, and cell surface expression of activation markers [CD80, CD86, CD40, and major histocompatibility complex (MHC) class I] was evaluated. PMG5 did cause a significant increase in CD40 expression, while soluble PAMAM G5 did not (Fig. 5A). CD40 (on DCs) plays a role in stimulating antigen-specific CD8^+^ T cell immune responses indirectly upon interaction with CD40L on CD4^+^ T cells or directly through interaction with CD40L on CD8^+ ^T cells (*40*). PMG5 did not cause a significant increase in CD80 (Fig. 5B), CD86 (Fig. 5C), or MHC class I (Fig. 5D) versus untreated BMDCs, while soluble PAMAM G5 significantly increased CD80 expression (Fig. 5B). In an effort to discern the type of immune response [T helper cell 1 (T_H_1) versus T_H_2] that may be promoted upon PMG5 interacting with BMDCs, the levels of secretion of two cytokines [interleukin-6 (IL-6) and interferon gamma-induced protein-10 (IP-10)] by BMDCs cultured with PMG5 were evaluated. While IL-4 is a potent T_H_2 cytokine promoting naïve T cells toward a T_H_2-biased phenotype, DCs do not secrete IL-4; thus, IL-6 was chosen as reports indicate that secretion of IL-6 from DCs causes secretion of IL-4 from T cells, steering them toward a T_H_2 phenotype (*41*, *42*). IP-10 (also known as CXCL10) was chosen because it has demonstrated effectiveness as a chemoattractant for T_H_1 cells and has been shown to be important for T_H_1-based antitumor activity (*43*–*45*). It was found that PMG5 did not cause significant increases in the secretion of either IP-10 or IL-6, in the presence or absence of Ad5-TRP2, while the Toll-like receptor 9 (TLR9) agonist, CpG ODN (a positive control), promoted significant increases in the secretion of both cytokines (Fig. 5, E and F).

### TLR activation by PMG5 and soluble PAMAM G5

Further investigation into the potential adjuvant properties of PMG5 was performed by testing the ability of PMG5 to stimulate a suite of TLRs as described in Materials and Methods. Results are summarized in fig. S3 and revealed that neither PMG5 nor soluble PAMAM G5 could stimulate any TLRs.

### In vitro evaluation of PM made with generations 3 and 4 PAMAM

Given the antitumor efficacy observed when mice were treated with Ad5-TRP2/PMG5, PM variants were made using different generations of PAMAM (generations 3 and 4, ethylenediamine core) to see whether other formulations could further improve therapeutic outcomes. Given the size-dependent uptake of PMG5 formulations, the particle characteristics of these PM formulations were also evaluated. The hydrodynamic diameter of the PM appeared to increase with decreasing generation number; i.e., PM made using generation 3 PAMAM (PMG3) had a hydrodynamic diameter of 231.68 ± 2.36 nm, while PM made using generation 4 PAMAM (PMG4) had a hydrodynamic diameter of 172.36 ± 3.01 nm (fig. S4D). The surface charges of PMG3 and PMG4 were +39.24 ± 0.56 and +42.6 ± 0.64 mV, respectively (fig. S4D). PMG4 and PMG3 also proved to be stable with small changes in size and surface charge after 30 days in Nanopure water at 37°C and room temperature (fig. S5, A and D). The PDI of both PMG3 and PMG4 appeared to be stable at room temperature for 40 days. However, for PMG4, incubating beyond 10 days at 37°C, the PDI progressively increased (fig. S5E). Evaluating the uptake of PMG3, PMG4, and PMG5 by BMDCs demonstrates that PMG5 is taken up to a significantly greater degree (as indicated by the increased MFI of BMDCs) than PMG3 (8.13 ± 0.63 versus 5.46 ± 0.67, *P* = 0.0002) and marginally more (but not statistically significant) than PMG4 (8.93 ± 0.63 versus 8.13 ± 0.30, *P* = 0.314) (fig. S6).

### Antitumor efficacy of PMG3, PMG4, and PMG5 in therapeutic Ad5-TRP2–vaccinated mice

**Fig. 6.**
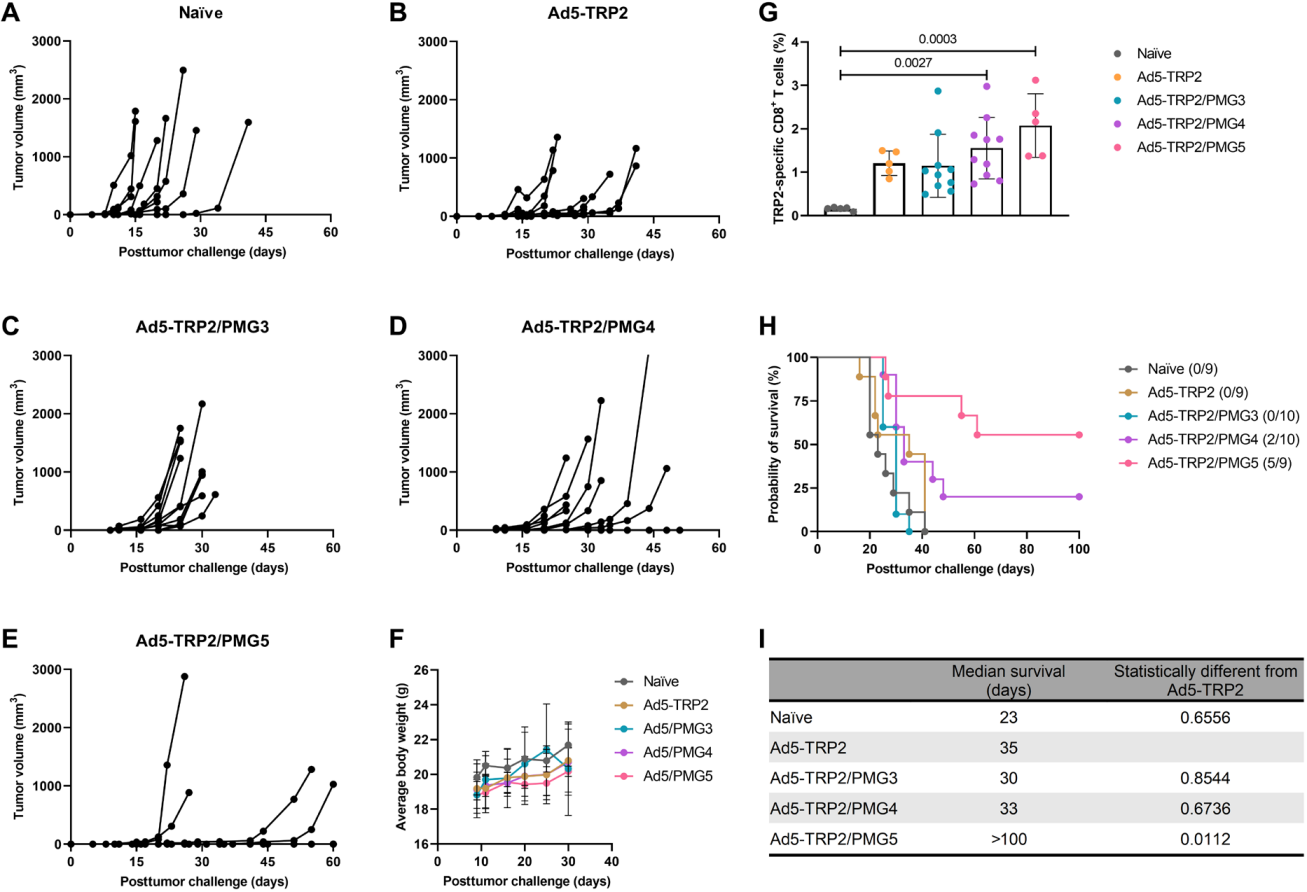
Therapeutic immunizations of C57BL/6J mice with Ad5-TRP2 and peritumoral administrations of PMG3, PMG4, or PMG5. (**A** to **E**) Tumor volume curves of mice treated with the indicated treatments. (**F**) Average body weight of mice. (**G**) Graph showing the percent of TRP2-specific CD8^+^ T cells in the peripheral blood (14 days after tumor challenge). (**H**) Survival curve of mice treated with different treatments after being challenged with tumors. Numbers in parentheses indicate the number (numerator) still alive at day 100. (**I**) Table showing the median survival of mice. Data are plotted as means ± SD. Probability as determined by one-way ANOVA with Tukey posttest.

The antitumor efficacy of PMG3 and PMG4 in therapeutically vaccinated mice was evaluated similar to how PMG5 was tested. The antitumor efficacy observed with Ad5-TRP2/PMG5 was greater than with Ad5-TRP2/PMG3 and Ad5-TRP2/PMG4 (Fig. 6, C to E). All mice treated with Ad5-TRP2/PMG3 (10 of 10 mice) succumbed to tumor burden by day 30 PTC, while 8 of 10 mice treated with Ad5-TRP2/PMG4 succumbed to tumor burden by day 45 PTC. This is in stark contrast to mice treated with Ad5-TRP2/PMG5 where delayed tumor growth was observed in mice that do develop tumors (four of nine) while five of nine mice did not develop tumors. This translated to mice treated with Ad5-TRP2/PMG3 or Ad5-TRP2/PMG4 having a median survival of 30 days and 33 days PTC, respectively, while for Ad5-TRP2/PMG5–treated mice, the median survival was greater than 100 days PTC (Fig. 6I). The survival percentages by the end of the study (day 60 PTC) for mice treated with Ad5-TRP2/PMG5, Ad5-TRP2/PMG4, or Ad5-TRP2/PMG3 were 66.6, 20, and 0%, respectively (Fig. 6H). The level of TRP2-specific CD3^+^ CD8^+^ T lymphocytes also progressively increased in mice treated with PM formulations made from increasing generations of PAMAM in vaccinated mice: Ad5-TRP2/PMG3 (1.147 ± 0.28%), Ad5-TRP2/PMG4 (1.554 ± 0.71%), and Ad5-TRP2/PMG5 (2.076 ± 0.73%) (Fig. 6G). This indicates that the antitumor effects of PMG5 in therapeutically vaccinated mice are unique to the PMG5 particle formulation and may be due to their size and/or charge, which differed significantly when compared to PMG3 and PMG4 (fig. S4).

### CD8^+^ T cell accumulation at the tumor site subsequent to PMG3, PMG4, and PMG5 administration to Ad5-TRP2–vaccinated mice

**Fig. 7.**
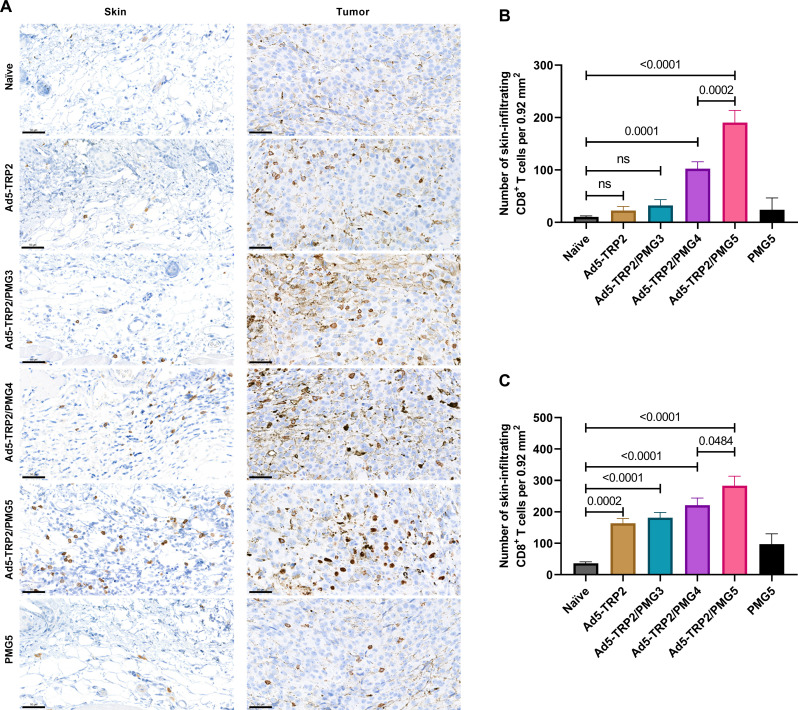
Accumulation of CD8^+^ T cells at the tumor site. Tumor-challenged mice were treated as indicated (and described in Materials and Methods). (**A**) On day 13 after vaccination, tumor and skin samples from mice (*n* = 3 per group) were stained for CD8 (scale bars, 50 μm), and the numbers are enumerated and compared: (**B**) skin and (**C**) tumor. CD8 expression is indicated by brown staining. Data are plotted as means ± SD.

Since it was likely that activated CD8^+^ T cells mediate the antitumor response observed with Ad5-TRP2/PMG5, we wished to test the hypothesis that PMG5 may promote enhanced infiltration of CD8^+^ T cells to the tumor site (i.e., skin peripheral to the tumor where the PM formulations were administered and/or intratumorally). Thus, 1 day after the last NP administration (day 13 after Ad5-TRP2 vaccination), mice were euthanized, and the relevant skin and tumor samples were harvested, fixed, sectioned, and stained for the presence of CD8^+^ T cells. Enumeration revealed that mice treated with Ad5-TRP2/PMG5 exhibited significantly enhanced levels of CD8^+^ T cells in both the peripheral skin and intratumorally compared to all other treatment groups (Fig. 7).

### Antitumor efficacy of PMG3, PMG4, and PMG5 in therapeutic Ad5-TRP2–vaccinated mice

**Fig. 8.**
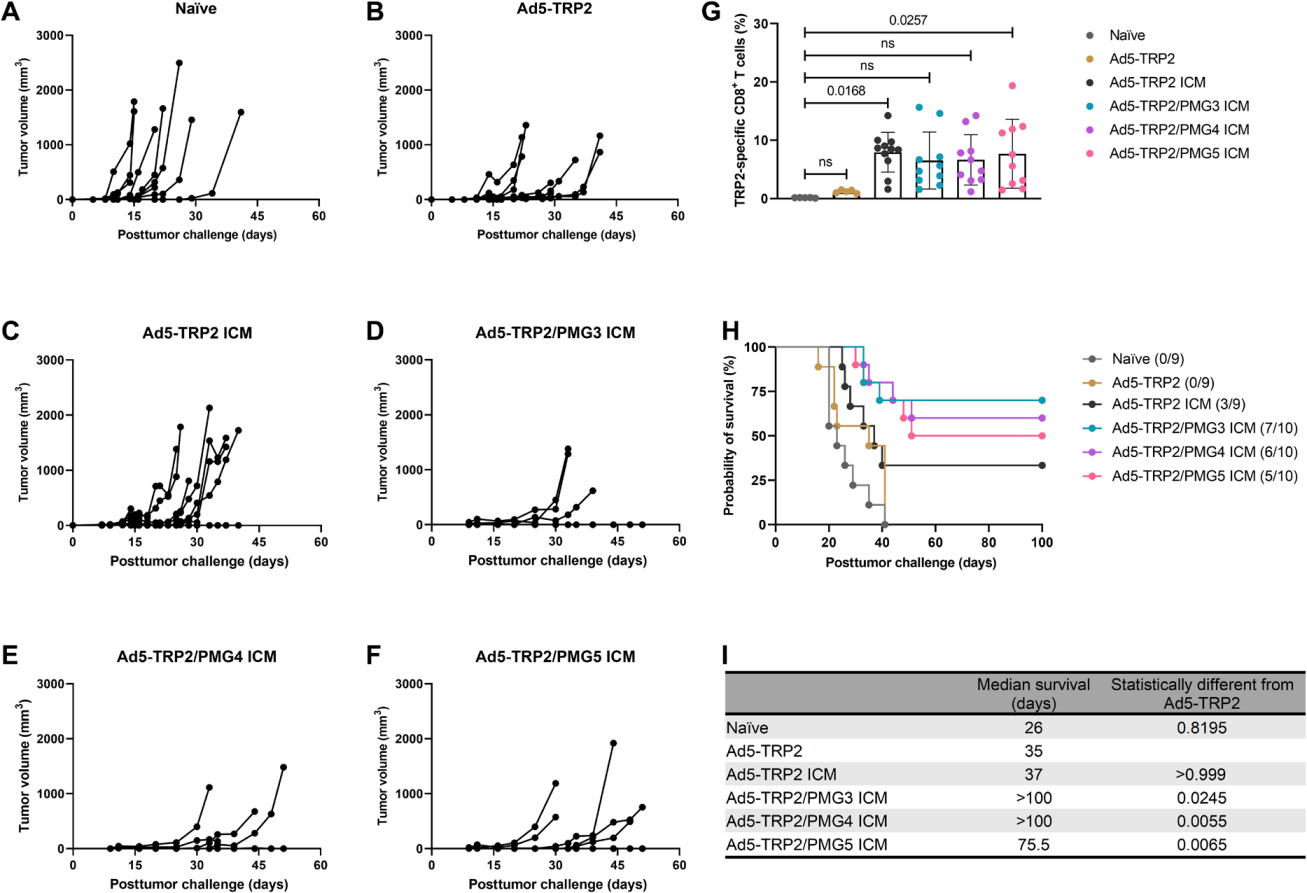
Effect of therapeutic immunizations of C57BL/6J mice with Ad5-TRP2 combined with systemic ICM and peritumoral administrations of PMG3, PMG4, or PMG5. (**A** to **F**) Tumor volume curves of mice treated with different designated treatments. (**G**) Graph showing the percent of TRP2-specific CD8^+^ T cells in the peripheral blood (14 days after tumor challenge). (**H**) Survival curve of mice treated with different treatments after being challenged with tumors. (**I**) Table showing the median survival of mice. Data are plotted as means ± SD. The numbers above the graphs refer to the probability as determined by one-way ANOVA with Tukey’s posttest.

Antibodies specific for immune checkpoint proteins, whether they be antagonist antibodies that block immunosuppressive pathways such as anti–PD-1 and anti–CTLA-4 or agonist antibodies that trigger T cell activation and proliferation such as anti–4-1BB, have demonstrated significant success in cancer immunotherapy either clinically or in preclinical settings, respectively. Given their dependence on tumor-specific T cells for efficacy and the demonstrated synergy between inhibiting T cell exhaustion by administering anti–PD-1 and promoting T cell survival and proliferation with anti–4-1BB (*46*–*49*), we evaluated the effects of this combination when combined with the Ad5-TRP2 cancer vaccine and PMG3, PMG4, or PMG5. Before launching into these studies, we first established that the combination of anti–PD-1 and anti–4-1BB was more efficient at promoting TRP2-specific immune responses and enhancing the survival of B16.F10-challenged mice when combined with Ad5-TRP2 compared to either immune checkpoint modulation (ICM) antibody alone (fig. S7). Mice vaccinated with Ad5-TRP2 and subsequently treated with anti–PD-1/anti–4-1BB demonstrated significantly higher levels of TRP2-specific CD3^+^CD8^+^ T lymphocytes in the peripheral blood (7.94 ± 3.41%) compared to Ad5-TRP2 alone; however, the addition of any of the PM formulations to the combination of Ad5-TRP2 and ICM did not result in any significant changes in levels of TRP2-specific CD3^+^CD8^+^ T lymphocytes when compared to Ad5-TRP2 + anti–PD-1/anti–4-1BB (Fig. 8G).

When compared to the survival of mice treated with Ad5-TRP2 plus either PMG3 or PMG4 (Fig. 6), there was a substantial improvement in survival when ICM was included (Fig. 8) such that the survival rates were similar to that obtained for mice treated with Ad5-TRP2/PMG4 alone (Fig. 6). The addition of ICM to the Ad5-TRP2/PMG5 group did not further enhance survival (Fig. 8). To elaborate, at 100 days PTC, mice treated with Ad5-TRP2/PMG3 ICM demonstrated 70% survival versus 0% survival for mice treated with Ad5-TRP2/PMG3, mice treated with Ad5-TRP2/PMG4 ICM demonstrated 60% survival versus 20% survival for mice treated with Ad5-TRP2/PMG4, and, lastly, mice treated with Ad5-TRP2/PMG5 ICM had 50% survival versus 55% survival for mice treated with Ad5-TRP2/PMG5. These findings did not reveal statistically significant differences between the survival curves of Ad5-TRP2/PMG3 ICM versus Ad5-TRP2/PMG4 ICM versus Ad5-TRP2/PMG5 ICM. The increase in antitumor efficacy (for Ad5-TRP2/PMG3 ICM and Ad5-TRP2/PMG4 ICM) versus Ad5-TRP2 ICM alone (as indicated by increased survival) occurred without a concomitant increase in levels of TRP2-specific CD3^+^CD8^+^ T lymphocytes in the blood: Ad5-TRP2/PMG3 ICM (6.543 ± 4.87%) and Ad5-TRP2/PMG4 ICM (6.651 ± 4.32%) versus Ad5-TRP2 ICM (7.9 ± 3.25%). This indicates that the effects of this increased antitumor efficacy may be due to the effects of the particles on the local tumor milieu that make the tumor cells more vulnerable to tumor cell killing by T cells but not through the promotion of T cell proliferation. Combining PM formulations and ICM in Ad5-TRP2–vaccinated mice did not result in any observable signs of toxicity to the mice as there was no obvious decrease in mice weights (fig. S8).

### Evaluating the effects of immune cell populations on the Ad5-TRP2/PMG5 and Ad5-TRP2/PMG5 ICM treatment groups

**Fig. 9.**
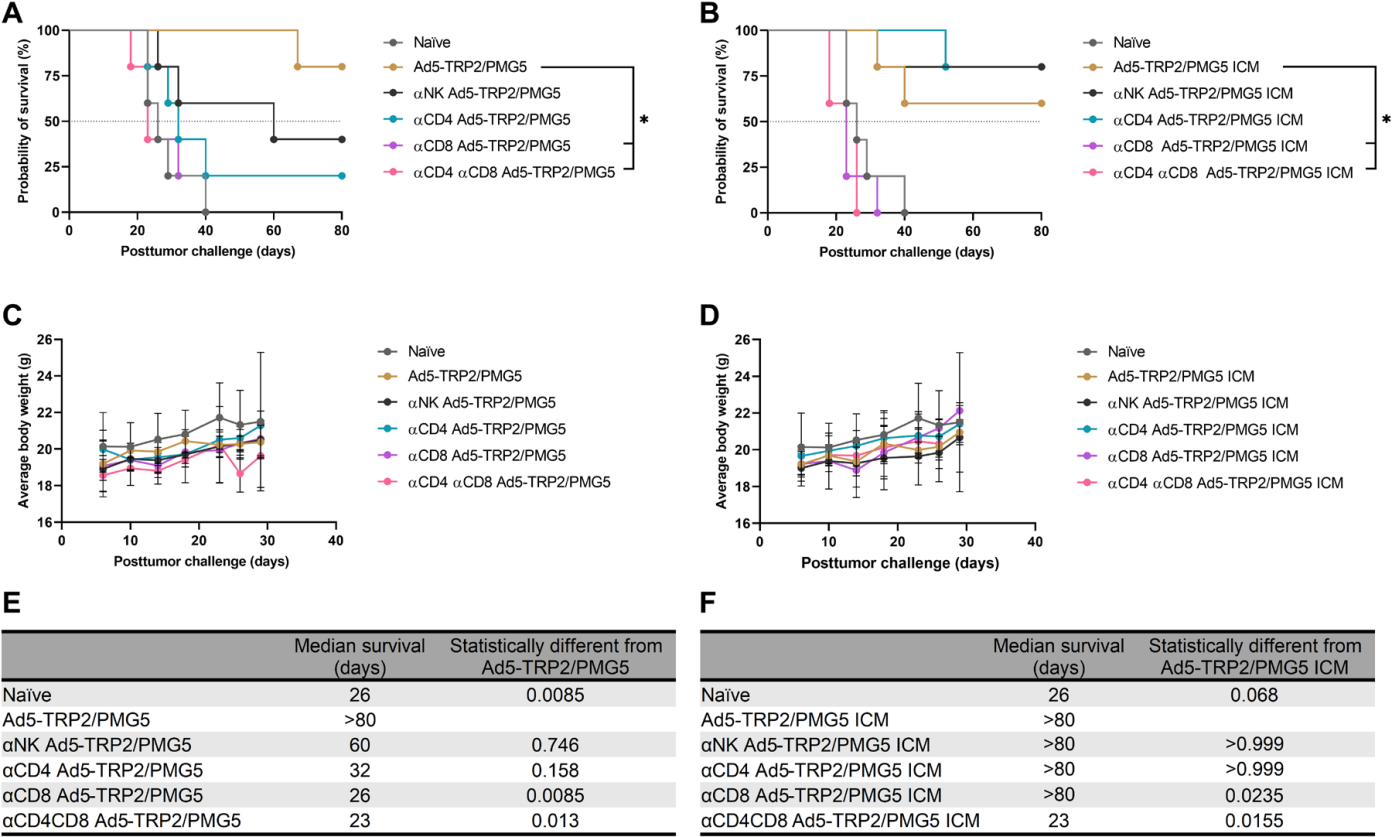
Evaluation of the effector immune cell population responsible for antitumor efficacy. (**A**) Survival curve of mice treated with Ad5-TRP2 PMG5 ± depletion of the indicated immune cell populations. *P < 0.05 compared to Ad5-TRP2/PMG5-treated group. (**B**) Survival curve of mice treated with Ad5-TRP2/PMG5 ICM ± depletion of the indicated immune cell populations. *P < 0.05 compared to Ad5-TRP2/PMG5 ICM-treated group. (**C**) Graph showing the average weight of mice treated with Ad5-TRP2 PMG5 ± depletion of the indicated immune cell populations over time. (**D**) Graph showing the average weight of mice treated with Ad5-TRP2/PMG5 ICM ± depletion of the indicated immune cell populations over time. (**E** and **F**) Tables showing the median survival of mice. Data are plotted as means ± SD. Statistical significance was determined using the log-rank test adjusted for multiple comparisons. *N* = 5 mice per group.

As thus far observed, PMG5 significantly increases the effects of the Ad5-TRP2 vaccine in a therapeutic setting, while other PM formulations have either no effect (PMG3) or a modest increase in survival (PMG4). However, when ICM is included, Ad5-TRP2/PMG5 ICM does not seem to provide an increase in efficacy versus Ad5-TRP2/PMG5, while ICM added to Ad5-TRP2/PMG3 or Ad5-TRP2/PMG4 increased the survival of each entity at least additively. In an effort to elucidate which cells are responsible for the efficacy of the Ad5-TRP2/PMG5 treatment group, these mice were depleted of select lymphocyte populations [natural killer (NK) cells, CD4^+^ T cells, CD8^+^ T cells, or CD4^+^/CD8^+^ T cells]. As a comparison, mice treated with Ad5-TRP2/PMG5 ICM were also depleted of the same select lymphocyte populations. Figure 9A demonstrates that for mice treated with Ad5-TRP2/PMG5, both adaptive immune cell populations (CD4^+^ T cells and CD8^+^ T cells) tested influenced the observed antitumor efficacy as indicated by decreased survival (although only CD8 T cell depletion resulted in statistically significant differences). Depleting NK cells showed a trend toward reducing the antitumor efficacy, but this decrease was not statistically significant from the Ad5-TRP2/PMG5 survival curve. At 80 days PTC, mice treated with Ad5-TRP2/PMG5 displayed 80% survival (four of five mice), depleting NK cells resulted in 40% survival (two of five mice), and depleting CD4^+^ T cells resulted in 20% survival (one of five mice). Depleting CD8^+^ T cells completely abrogated the antitumor efficacy of Ad5-TRP2/PMG5 treatment with all mice succumbing to tumors by day 40 PTC. In contrast, Ad5-TRP2/PMG5 ICM–treated mice appeared to have relied exclusively on CD8^+^ T cells mediating antitumor efficacy. On day 80 PTC, mice treated with Ad5-TRP2/PMG5 ICM demonstrated 60% survival (three of five mice), while depleting CD4^+^ T cells or NK cells resulted in 80% survival (four of five mice). However, depleting CD8^+^ T cells resulted in five of five mice succumbing to tumors by day 30, and depleting both CD8 and CD4 resulted in five of five mice succumbing to tumors by day 26 PTC. Taking these results into account, it points to Ad5-TRP2/PMG5 mice possibly relying on both the adaptive and innate immune system to elicit its antitumor efficacy, and supplementing Ad5-TRP2/PMG5 treatment with ICM shifts the response to being solely reliant on CD8^+^ T cells mediating an effector response. The tumor volumes for each mouse from each treatment were also recorded and are shown in fig. S9.

## DISCUSSION

Cancer vaccines hold enormous potential for the treatment of intractable tumors such as late-stage melanoma. However, despite promising results in preclinical settings for cancer vaccines per se, their effectiveness in the clinic has not been demonstrated, especially when compared to other cancer immunotherapeutic modalities such as immune checkpoint therapy and adoptive T cell therapy (*50*). This is likely due, in part, to the immunosuppressive nature of the TME, and thus, tackling such immunosuppressive obstacles on potentially more than one front in combination with cancer vaccine administration may provide synergistic therapeutic benefit. Several lines of evidence support the feasibility of this type of approach. For instance, it has been previously demonstrated that local delivery of adjuvants such as CpG (type B) initiates in situ immunization, and combining anti-OX40 ICM with intratumoral CpG (type B) administration abrogates tumor growth and protects mice from tumor rechallenge (*51*). Previous studies by our group have demonstrated that intratumoral administration of CpG (type B) in combination with therapeutic vaccination with an adenovirus cancer vaccine encoding a model tumor antigen increases its antitumor efficacy, resulting in increased survival. The administration of CpG was shown to increase the levels of tumor-specific T cells and reduce the levels of regulatory T cells within the tumor (*22*). Recently, it has been shown that intratumoral administration of CMP-001, a virus-like particle encapsulating CpG (type A), in combination with systemic administration of immune checkpoint blockade therapy provided increased protection in mice versus mice treated with anti–PD-1 alone (*52*). These studies indicate that (i) delivery of adjuvants to the site of the tumor can at least partially abrogate the immunosuppressive phenotype of the tumor, thus enabling the function of tumor-specific CTLs, including those generated by a cancer vaccine; (ii) combining this approach with ICM therapy (i.e., agonist antibodies and/or antagonist antibodies) and therefore dampening the immunosuppressive potency within the TME has the potential to synergistically increase the antitumor efficacy of therapeutic cancer vaccines. Thus, we sought to bring these approaches together in a multipronged strategy to generate optimal tumor-specific immune responses in a therapeutically relevant tumor model using a murine melanoma cancer vaccine. Late-stage melanoma is a major health concern with a 9 to 19% 5-year survival rate and has shown to be responsive to immunotherapies such as ICM in clinical settings (*53*). Here, we used an attenuated Ad5 encoding for a TAA, TRP2, as the cancer vaccine in a poorly immunogenic B16.F10 murine melanoma model. TSAs admittedly may be better at generating an immune response; however, TSAs lack the possibility of being prepared on a large standardized scale as they are often patient or even tumor specific, and there is still the issue of the immunosuppressive TME (*18*). Also, given the lack of immunogenicity associated with TAAs, a cancer vaccine strategy that generates a substantial immune response against TAAs will more than likely generate the same, if not greater, response should TSAs be implemented instead. This approach using a poorly immunogenic TAA to treat a murine melanoma model, which has been noted to be poorly responsive to immunotherapy, will serve as a rigorous testing platform that mimics the clinical situation with respect to a lack of significant antitumor efficacy being demonstrated when the cancer vaccine is administered alone.

The prophylactic studies presented demonstrated the capability of the Ad5-TRP2 vaccine to trigger a detectable TRP2-specific CD8^+^ T lymphocyte response and that the resulting immune response had antitumor activity as it led to significant protection of mice challenged with B16.F10 cells (Fig. 3). On the basis of previous studies, this protection is likely to have been mediated by CD4^+^ T lymphocytes and CD8^+^ T lymphocytes contributing to the priming and effector stages, respectively (*54*). In our experience, B16.F10 cells, as evidenced by the tumor volume graphs, grow very aggressively, and therefore, we chose an early time point (day 1 PTC) to commence the Ad5-TRP2 vaccination treatment in the therapeutic studies. Despite being able to protect mice from B16.F10 tumor challenge in a prophylactic setting, Ad5-TRP2 was unable to significantly improve mouse survival in a therapeutic setting (Fig. 4H). To improve the antitumor efficacy of Ad5-TRP2, we delivered PMG5 to the site of tumor challenge (peripherally), which was contralateral to the Ad5-TRP2 vaccine. PMG5 is a novel cationic nanoscale adjuvant that has been developed in our group to serve as a platform to increase the efficacy of immunotherapeutic modalities. We hypothesized that PMG5 may act to increase the efficacy of the Ad5-TRP2 vaccine by stimulating a local inflammatory environment, resulting in the increased accumulation of activated CD8^+^ T cells to the tumor site and/or creating a microenvironment that favors CD8^+^ T cell activity. Thus, PMG5 was delivered on days 7 to 12 after vaccination since this would allow time for the Ad5-TRP2 vaccine to stimulate the activation of TRP2-specific CD8^+^ T cells and their subsequent release into the peripheral blood where they would circulate in search of signs of inflammation. Characterization of PMG5 indicates that it is thermally stable below 50°C and stable in an aqueous solution for at least 10 days without significant change in size and surface charge (fig. S1). The combination of PMG5 and Ad5-TRP2 as a therapeutic treatment in B16.F10-challenged mice demonstrated significantly higher survival (compared to untreated mice) as well as reduced tumor burden and protection from future tumor challenges, implying that immunological memory was generated. These effects were not noted in mice receiving PMG5 alone. An increase in the efficacy of the Ad5-TRP2 vaccine was not noted when soluble CpG (type B) was used as a treatment instead of PMG5, as evidenced by lower levels of TRP2-specific CD3^+^CD8^+^ T lymphocytes (Fig. 4I) and lower median survival (Fig. 4G). To gain some insight into the potential adjuvant properties of PMG5, in vitro BMDC activation studies were performed where it was observed that PMG5 significantly up-regulated surface CD40 expression by DCs albeit only marginally (Fig. 5A). The potential effect of up-regulating CD40 may be to place less reliance on CD4^+^ T cells in activating or promoting survival of CD8^+^ T cells at the tumor-draining lymph node or at the tumor locale; however, without further investigation, the importance of CD40 in these studies can only be speculated upon. The lack of TLR agonist properties of PMG5 (and soluble PAMAM G5) (fig. S3) and the lack of substantial CD80, CD86, and IP-10 up-regulation by PMG5 (Fig. 5) in vitro suggest that there are likely yet to be determined innate immune molecular pathways through which PMG5 may act to achieve its adjuvant effect in vivo.

We additionally demonstrated that this antitumor efficacy was unique to this formulation, and particles of the different polymer compositions (PMG3 and PMG4) did not elicit similar antitumor efficacy when administered in combination with Ad5-TRP2 (Fig. 6). Together, this demonstrates that PMG5 can serve as a potent adjuvant in vivo since it can increase the efficacy of a cancer vaccine. As stated above, the molecular mechanism by which PMG5 promotes its adjuvant effect is not definitively understood; however, from our investigative in vivo data into the cellular mechanisms (Fig. 7), it was revealed that PMG5 could significantly enhance (compared to all other treatment groups) the accumulation of CD8^+^ T cells at the site of the tumor (both intratumorally and peritumorally at the site of PMG5 administration). It is well known that, upon activation, T lymphocytes will circulate in the peripheral blood until encountering a site of inflammation, which they will then infiltrate (*55*). Thus, it is likely that PMG5 has promoted the infiltration of TRP2-specific CD8^+^ T cells to a greater degree than PMG3 and PMG4 by creating a more favorable inflammatory environment conducive to T cell infiltration and therefore potentially explaining the therapeutic superiority of PMG5. It has been previously demonstrated that TRP2_180–188_-specific CD3^+^CD8^+^ T lymphocytes activated by adenoviral-based vaccines (encoding TRP2) are responsible for melanocyte-specific killing and therefore very likely to have played a role in the killing of the B16.F10 cells in the therapeutic model used here (*56*). Thus, the increase in efficacy when PMG5 was administered was likely due to the effect that PMG5 had on the TME, allowing for an increase in cytotoxicity and/or proliferation of TRP2_180–188_-specific CD3^+^CD8^+^ T lymphocytes. In the absence of a tumor, there was no significant change in the levels of TRP2-specific CD3^+^CD8^+^ T lymphocytes in the peripheral blood lymphocyte (PBL), suggesting that PMG5 had an influence on the TME rather than directly affecting the efficacy of the cancer vaccine (fig. S10). Another possible, and not necessarily mutually exclusive, reason for the differences in therapeutic efficacy between PMG5 and PMG4 and PMG3 may be due to the difference in the size of these particle formulations (fig. S4) and their resultant differences in the ability to traffic to the lymph nodes and interact with immune cell populations there (*51*, *57*).

Combining Ad5-TRP2/PMG3 or Ad5-TRP2/PMG4 with ICM leads to an increase, albeit not statistically significant, in the median survival of mice versus Ad5-TRP2/ICM alone (Fig. 8H). The trending increase in the overall survival of mice treated with this multipronged therapy occurred without a concomitant increase in the levels of TRP2-specific CD3^+^CD8^+^ T lymphocytes in the peripheral blood, indicating that the efficacy was likely due to the effects that PM formulations had on the TME and/or its surroundings (Fig. 8G). Combining Ad5-TRP2/PMG5 with ICM also did not result in an increase in TRP2-specific CD3^+^CD8^+^ T lymphocytes in the peripheral blood versus Ad5-TRP2/ICM (Fig. 8G); however, unlike the findings for Ad5-TRP2/PMG3 and Ad5-TRP2/PMG4, there was no improvement in the overall survival of mice treated with Ad5-TRP2/PMG5 ICM versus Ad5-TRP2/PMG5 (Fig. 6H, versus Fig. 8H). The reasons for the discrepancy in findings when comparing PMG5 to either PMG3 or PMG4 are not readily apparent; however, the differences in size and surface charge observed cannot be ruled out as a contributing factor. It is possible that PMG5 affected the TME in such a way that the addition of ICM (which can also affect the TME) is redundant. Further studies will be required to determine whether this, or some other explanation, is valid.

The lack of antitumor efficacy of PMG5 in unvaccinated mice indicates that the efficacy of the Ad5-TRP2/PMG5 treatment was not due to tumor cell death directly induced by PMG5 but possibly an immunological effect relating to the innate arm of the immune system that creates a favorable environment for CTL activity. Depletion studies tentatively, though not conclusively, indicate this where Ad5-TRP2/PMG5–treated mice appeared to have a broader reliance on a diverse array of immune cells tested (NK cells and CD4^+^ and CD8^+^ T cells), while Ad5-TRP2/PMG5 ICM–treated mice relied predominantly on CD8^+^ T cells to mediate an antitumor effector response. It is possible that because of this reliance on multiple cell types (in Ad5-TRP2/PMG5–treated mice), individually depleting one of these cell types (e.g., NK cells), while having an apparent impact on survival, did not attain statistical significance because of the contribution of other cell types (e.g., CD4^+^ and CD8^+^ T cells). Upon the addition of ICM, we propose that the tumor-specific CD8^+^ T cells no longer required assistance from CD4^+^ T cells and NK cells to mediate the antitumor effect and that ICM was providing (or blocking) the signals to CD8^+^ T cells required to promote an optimal effector response. PMG5 also demonstrated increased uptake in BMDCs versus PMG4 and PMG3 (fig. S6), indicating the ability of PMG5 to be taken up by immune cell populations such as DCs and elicit downstream signals that are possibly similar to, or overlap with, the signaling prompted by ICM, thus potentially rendering the treatments redundant; while PMG3 and PMG4 potentially lack the capacity to provide these downstream signals that affect immune efficacy given their decreased uptake by BMDCs.

It is important to put the results obtained into the broader context of cancer immunotherapy. We have demonstrated that combining a single administration of an adenovirus-based cancer vaccine with three administrations of PMG5 provides durable antitumor immune responses and protects mice from tumor rechallenge. Previous preclinical vaccination studies in mice have demonstrated the ability of Ad5-TRP2 and Ad5-mTRP2 to protect against B16.F10 metastases (*55*, *58*); however, their ability to effectively combat solid B16.F10 tumors in a therapeutic setting has rarely been investigated, possibly because B16.F10 solid tumors are notoriously difficult to eradicate with cancer vaccines in general. Successful attempts have been recorded using multiple doses and high titers of Ad2-mTRP2 or using a multipronged non–viral-based vaccine regimen (*59*, *60*). Additional studies have demonstrated the efficacy of combining immune checkpoint agonists and antagonists (*48*). To the best of the authors’ knowledge, this is the first demonstration of combining a local administration of a cationic nanoparticulate adjuvant formulation and a cancer vaccine with positive results. These results pave the way for further studies to investigate the mechanism of action of PMG5 when used in combination with TSA- or TAA-based cancer vaccines.

In summary, we have successfully synthesized and characterized a previously unexplored cationic NP formulation using PLGA and PAMAM (PM). We have also demonstrated that by combining a single administration of Ad5-TRP2 with subsequent peritumoral administrations of PMG5, we can significantly increase the efficacy of the Ad5-TRP2 cancer vaccine in treating the murine melanoma model. This treatment regimen not only stops tumor growth but also protects mice from subsequent tumor challenges. This is evident from the elevated TRP2-specific CD8^+^ T cell response, the significant increase in median survival, and prolonged survival of mice receiving this treatment regimen. We have also shown that combining underperforming PM formulations with ICM results in a significant increase in the efficacy of the treatments. This demonstrates that this formulation can be combined with other immunotherapy modalities to yield additional antitumor efficacies. Further studies are required to define specific molecular requirements such as important signaling pathways, cytokines, and surface receptors as well as important cell populations that PMG5 may be directly affecting. Ultimately, PMG5, or an optimized version thereof, may prove of benefit in the clinic when combined with a therapeutic cancer vaccine, particularly for patients with melanoma where the mutation rate (and therefore antigenicity) is high and where the lesions are readily accessible.

## MATERIALS AND METHODS

### Animals

All animal experiments involved 6- to 8-week-old, female C57BL/6J mice that were purchased from the Jackson Laboratory (Bar Harbor, ME) and housed in The University of Iowa animal care facility for a minimum of 1 week before use. All mice were maintained in filtered cages. All animal experiments were carried out in accordance with guidelines and regulations approved by The University of Iowa Institutional Animal Care and Use Committee.

### Adenovirus synthesis

The attenuated Ad5 used in these studies were manufactured by the Viral Vector Core (The University of Iowa, Carver College of Medicine, Iowa City, IA) using a method previously described (*61*). The viral DNA construct was engineered to express human TRP2 upon transduction of living cells. All Ad5 had a portion of the adenoviral genome deleted that included the left-hand terminal repeat, the packaging signal, and E1A and E1B sequences, rendering the virus replication deficient.

### PLGA/PAMAM NP (PMG5, PMG4, and PMG3) formulations

PLGA PAMAM NPs (PM) were fabricated using a modified nanoprecipitation method. Briefly, 50 mg of 50:50 PLGA (Resomer RG 502, Evonik, Parsippany, NJ) was dissolved in 5 ml of acetone. Then, 125 μl of PAMAM (ethylenediamine core; generation 5, 4, or 3; purchased from Sigma-Aldrich, St. Louis, MO) was added to make a resulting PAMAM solution at a concentration of 0.125% (w/v). PLGA/PAMAM solution was added dropwise from a syringe needle (G26) (without applied pressure, i.e., gravity dependent) into a 0.1% (w/v) polyvinyl alcohol aqueous solution [molecular weight (MW) of 9000 to 10,000 g/mol; Sigma-Aldrich] under stirring. Following this, instantaneously formed PMs were stirred in a fume hood for 30 min. PMs were then transferred to a rotary evaporator (Rotavapor R-300, Buchi, Switzerland), which was set to 50 mbar at 50 rpm for 4 hours to evaporate organic solvents. Then, PMs were washed with sterilized deionized water in Amicon Ultra centrifuge tubes (100,000 MWCO) four times by centrifuging at 500*g* for 1 hour. PMs were then frozen in a 10% sucrose solution at −80°C for 24 hours and then lyophilized for 48 hours using a freeze dryer (Labconco, FreeZone, 4.5 liters, Kansas City, MO). Last, dry PMs were collected and stored in sealed containers until use. To evaluate the impact of involving the lyophilization process and adding sucrose to the formulation, two other different formulations of PMs were prepared; one was freshly prepared (i.e., no freezing or lyophilization), while the other batch was prepared and lyophilized in the absence of a cryoprotectant (i.e., containing no sucrose). PMs synthesized using PAMAM (ethylenediamine core, generation 5) were designated as PMG5, and those synthesized using PAMAM (ethylenediamine core, generations 3 and 4) were designated as PMG3 and PMG4, respectively. Note that we also attempted to manufacture PMs with PAMAM generations 6 and 7 using the same method used to generate PMG3, PMG4, and PMG5; however, we found that both generations 6 and 7 precipitated in the organic phase (acetone) during manufacture and failed to efficiently incorporate into the resultant NPs, as indicated by their negative surface charge. Thus, no further investigation of these NPs was performed.

### Characterization of PM formulations

PMG5 were imaged using TEM (JEOL, JEM-1230, Tokyo, Japan). Briefly, lyophilized PMG5 (0.1 mg/ml) was added to 1.5% (v/v) sterile-filtered phosphotungstic acid at a ratio of 1:1. The resulting solution was placed on carbon-coated TEM grids and imaged to analyze the morphology of PMG5. PMG5 were further characterized by assessing the average hydrodynamic diameter, PDI, and net surface charge (zeta potential) using a Zetasizer Nano ZS (Malvern Panalytical, Malvern, UK) as previously described (*62*). In addition, the shape and surface morphology of PMG5 were further examined using a Hitachi SEM (Hitachi High-Technologies, Schaumburg, IL) (*63*). PLGA, PAMAM, and PMG5 were also characterized for heat flow properties by analyzing the thermograms obtained from the differential scanning calorimeter (DSC Q20, TA Instruments, New Castle, DE) equipped with a refrigerated cooling system (RCS90) (TA Instruments, New Castle, DE), as previously described (*64*). All samples were sealed in standard aluminum sample pans covered with lids. An empty sealed aluminum pan covered with a lid was used as a reference. Pure dry nitrogen (set at a flow rate of 40 ml/min) was used as a purge gas. Samples were heated from 0° to 100°C at a heating rate of 5°C/min.

### Degradation studies

In this experiment, 5 mg of lyophilized PMG3, PMG4, and PMG5 was dispersed in 5 ml of Nanopure water. The resulting solutions were placed at room temperature or 37°C and agitated at 300 rpm for 42 days. Samples were taken at various time points (every day for 3 days, then every week for 2 weeks, and then every 2 weeks), and the size, zeta potential, and PDI were recorded using the Zetasizer Nano ZS (*62*).

### Evaluation of the effect of lyophilization

Because of the intrinsic fluorescence of PAMAM, the cellular uptake behavior of PMG5 could be directly analyzed by fluorescence microscopy and flow cytometry, without additional fluorescence labeling (*65*). Here, quantitative and qualitative cellular uptake of PMG5 by BMDCs was studied. For tracking the in vitro cellular uptake of PMG5, BMDCs were incubated with 0.12 mg of differently prepared PMG5 as follows: (i) nonlyophilized PMG5 (freshly prepared), (ii) lyophilized PMG5 in the presence of sucrose, (iii) lyophilized PMG5 without sucrose, and (iv) untreated BMDCs as a control. Cells were then collected (without using trypsin; instead, vigorous flushing was implemented) and centrifuged (230*g*) for 5 min at 4°C. All cell samples were run through a BD FACScan flow cytometer (Becton Dickinson, Franklin Lakes, NJ) in triplicate, and data were analyzed with FlowJo software (Tree Star, Ashland, OR).

### Harvesting BMDCs

BMDCs were generated following a procedure previously published (*4*, *66*, *67*). In brief, femurs and tibia were harvested from C57BL/6J mice, and then the bone marrow was flushed with RPMI 1640 cell culture media supplemented with 0.01 M Hepes buffer (Gibco, Thermo Fisher Scientific, Waltham, MA), 1 mM sodium pyruvate (Gibco, Thermo Fisher Scientific), 1× GlutaMAX (Gibco, Thermo Fisher Scientific), 50 mM 2-mercaptoethanol (Sigma-Aldrich), gentamycin sulfate (0.5 mg/ml; IBI Scientific, Dubuque, IA), and 10% fetal bovine serum (Atlanta Biologicals, Flowery Branch, GA). Bone marrow cells were then counted and seeded at a density of 2 × 10^6^ cells in a 10-ml complete RPMI medium with GM-CSF (20 ng/ml) (Peprotech, Rocky Hill, NJ) at 37°C with 5% CO_2_ in bacteriological petri dishes. On day 3 (72 hours after seeding), 10 ml of RPMI complete media with GM-CSF (20 ng/ml) was added. On days 6 and 8, 10 ml of cell culture supernatant was harvested and spun at 230*g*, old media (supernatant) were aspirated, and 10 ml of fresh media with GM-CSF (20 ng/ml) was added. On day 10, cells were transferred to 12-well tissue culture–grade plates and seeded at a density of 1 × 10^5^ cells per well and allowed to equilibrate for at least 4 to 6 hours before incubating with experimental groups.

### PM uptake by BMDCs

BMDCs were incubated with four different doses (0.06, 0.12, 0.18, and 0.24 mg) of lyophilized PMG5 with sucrose to assess the effect of the dose on the uptake capacity. After incubation for 48 hours, cells were collected, and samples were acquired using a FACScan flow cytometer. BMDCs were initially seeded in a 12-well plate in media-containing serum, as described above. Before adding the PMG5, the media were removed, and fresh serum-free media were added. This was followed by adding the PMG5 (lyophilized with sucrose) to the BMDCs. Cells were then collected and analyzed using a FACScan flow cytometer and FlowJo software. A similar procedure was followed for PMG3 and PMG4 except that only one dose of the PM formulation (0.24 mg) was used.

### Mechanistic study of the uptake of PMG5 by BMDCs

This experiment was performed to study the mechanism(s) of uptake of PMG5 by BMDCs using inhibitors of endocytosis pathways. In general, endocytosis is an energy-dependent process that can be delineated as clathrin-mediated endocytosis, caveolae-mediated endocytosis, and macropinocytosis. In this study, BMDCs were incubated with different endocytosis pathway inhibitors including (i) sucrose to inhibit clathrin-mediated endocytosis (400 μM; added to the cells 1 hour before treating with PMG5), (ii) MβCD (Sigma-Aldrich) to inhibit caveolae-dependent endocytosis (1 mM; added to the cells 1 hour before treating with PMG5), and (iii) amiloride (Sigma-Aldrich) to inhibit macropinocytosis (2 mM; added to the cells 10 min before treating with PMG5). This was followed by adding the PMG5 (0.12 mg) and incubating it with cells for 3 hours. BMDCs were then harvested, and the data were acquired and analyzed using a FACScan flow cytometer and FlowJo software.

### Cytotoxicity study

BMDCs were seeded at a density of 1 × 10^4^ cells per well in a 96-well plate in 180 μl per well of complete RPMI 1640 cell culture media. After incubation for a few hours, cells were treated with an increasing concentration of PMG5 (50, 100, 200, 400, 800, and 1600 μg/ml). Also, corresponding concentrations of soluble PAMAM G5 (6.25, 12.50, 25, 50, 100, and 200 μg/ml) were tested. Plates were then incubated at 37°C with 5% CO_2_ for 48 hours. Next, Cell Titer 96 aqueous MTS reagent [3-(4,5-dimethylthiazol-2-yl)-2,5 diphenyltetrazolium bromide, purchased from Promega Corporation, San Luis Obispo, CA] was added to the cells following the manufacturer’s instructions (*63*, *68*–*70*). After incubation for 2 hours, cell viability (%) was calculated on the basis of the absorbance measured at 490 nm using a SpectraMax Plus spectrophotometer (Molecular Devices, San Jose, CA).

### BMDC activation studies

In this experiment, BMDCs were divided into separate treatment groups including naïve (untreated BMDCs), Ad5-TRP2 (BMDCs incubated with 1 × 10^8^ PFUs of Ad5-TRP2), PMG5 (BMDCs incubated with 1.6 mg of PMG5), CpG (BMDCs incubated with 50 μg of soluble CpG B 1826), and soluble G5 (BMDCs incubated with 12.5 μg of soluble G5). Cells were incubated with the indicated treatments for 2 days in 2 ml of complete media (*4*). The amounts of PMG5 and soluble PAMAM G5 added to the BMDCs were at subtoxic levels as determined by the cytotoxicity assay described above (see fig. S11). Supernatants from the cells were then collected and stored for later use in Luminex multiplex cytokine assays (*71*), and BMDCs were then flushed from the plate with ice-cold 1× Dulbecco’s phosphate-buffered saline (DPBS; Gibco, Thermo Fisher Scientific). BMDCs were collected, washed with ice-cold fluorescence-activated cell sorting (FACS) buffer [1× DPBS, 0.1% sodium azide, and 5% fetal calf serum (FCS)], and transferred to a 96-well U-bottom plate (Cellstar, Greiner). Cells were then incubated with a 1/100 dilution of anti-mouse CD16/CD32 (clone 93, Invitrogen) for 15 min on ice. BMDCs were then incubated with one or more of the following antibodies: anti-mouse CD11c fluorescein isothiocyanate (FITC) (clone N418, Invitrogen), anti-mouse CD40 APC (clone 3/23, BioLegend, San Diego, CA), anti-mouse CD80 APC (16-10A1), anti-mouse CD86 APC (20-0862, Tonbo Biosciences, San Diego, CA), and anti-mouse MHC class I (H-2Kb) PE (AF6-88.5.5.3, Invitrogen, Waltham, MA), for 30 min on ice in the dark (*72*). BMDCs were then washed twice with ice-cold FACS buffer and resuspended in 100 μl of Cytofix buffer (BD Biosciences, Franklin Lakes, NJ) and allowed to incubate for 10 min on ice in the dark. Next, 100 μl of ice-cold 1× Perm/Wash solution (BD Biosciences) was added to each well, and then cells were centrifuged at 660*g*, resuspended in ice-cold FACS buffer, and stored sealed at 4°C until they were acquired using the FACScan flow cytometer and analyzed using FlowJo software.

### Mouse TLR agonist screening

TLR stimulation was tested by assessing nuclear factor κB (NF-κB) activation in the TLR-expressing cell lines (InvivoGen, San Diego, CA). The activity of the test samples was tested on eight different mouse TLRs (TLR2, TLR3, TLR4, TLR5, TLR7, TLR8, TLR9, and TLR13) as potential agonists. The activity of the test samples is tested at one concentration and compared to control ligands including the following: heat-killed *Listeria monocytogenes* at 1 × 10^8^ cells/ml (HEK-Blue mTLR2), poly(I:C) HMW at 1 μg/ml (HEK-Blue mTLR3), *Escherichia coli* K12 lipopolysaccharide at 100 ng/ml (HEK-Blue mTLR4), *Salmonella typhimurium flagellin* at 100 ng/ml (HEK-Blue mTLR5), CL307 at 1 μg/ml (HEK-Blue mTLR7), TL8-506 at 1 μg/ml (HEK-Blue mTLR8), CpG ODN 1826 at 100 ng/ml (HEK-Blue mTLR9), and ORNSa19 at 200 ng/ml (HEK-Blue mTLR13). This assay was performed in triplicate. The secreted embryonic alkaline phosphatase (SEAP) reporter was under the control of a promoter inducible by the transcription factor NF-κB. This reporter gene allows the monitoring of signaling through the TLR, based on the activation of NF-κB. In a 96-well plate (200 μl in total volume), containing the appropriate cells, 20 μl of the test sample (soluble PAMAM G5 and PMG5) or the positive control ligand was added to the wells. A low, relatively nontoxic concentration of soluble PAMAM G5 (6.25 μg/ml) was used. Also, a higher concentration of soluble PAMAM G5 (100 μg/ml) and its corresponding PMG5 (800 μg/ml) were tested. The media added to the wells is designed for the detection of NF-κB–induced SEAP expression. After a 16- to 24-hour incubation, the optical density is read at 650 nm on a Molecular Devices SpectraMax 340PC absorbance detector.

### Prophylactic B16.F10 model

C57BL/6J female mice (*n* = 5 per treatment group) were first vaccinated subcutaneously in the right-hand side rear dorsal flank with the indicated dose of Ad5-TRP2. On day 14 after vaccination, mice were submandibular bled, and the harvested PBLs were stained for TRP2-specific CD8^+^ T cells using the method described below. Also, on day 14 after vaccination, the mice were challenged subcutaneously with 1 × 10^5^ live B16.F10 cells [in 100 μl of serum-free Dulbecco’s modified Eagle’s medium (DMEM)] on the left-hand side rear dorsal flank (contralateral to the vaccination). Mice were monitored for tumor size using calipers, and tumor volume was determined on the basis of the assumption that the tumors were ellipsoid in shape: [*V* = (diameter 1 × diameter 2 × height) × (π/6)]. Mice were humanely euthanized upon reaching endpoint criteria, which included having a tumor that had reached 20 mm in length or width or 10 mm in height. The experiment was terminated on day 55 PTC.

### Tumor challenge and treatment protocol (therapeutic B16.F10 model)

C57BL/6J mice were randomly divided into 12 groups at 9 to 10 mice per group, as described in Table 1. Mice were then challenged on the dorsal right flank with 2 × 10^5^ live B16.F10 cells in 100 μl of serum-free DMEM complete media. The following day, mice designated to receive Ad5-TRP2 were given a single dose contralaterally at 1 × 10^8^ PFUs. On days 8, 11, and 13 PTC, mice were given their designated treatment groups under anesthesia at the site of tumor inoculation on the right dorsal flank of the mouse. Tumor volumes and mice weights were recorded every 2 to 3 days. At 60 days after initial tumor challenge, mice that did not develop tumors initially were rechallenged with 2 × 10^5^ cells on the dorsal right flank. On days 8, 11, 13, 16, and 18 PTC, select groups were administered with αPD1 (100 μg per mouse per administration) and/or α4-1BB (intraperitoneally at 100 μg per mouse per administration) to provide ICM. Endpoint criteria were met when tumors reached 20 mm in length or width or 10 mm in height. Tumors were assumed to be ellipsoid in shape, and volumes were recorded by measuring the length, width, and height with calipers and calculated using the formula

$$\text{Volume}=(\text{length}\times \text{width}\times \text{height})\times \frac{\pi }{6}$$

**Table 1.** List of the experimental groups used in animal studies. α, anti.

**Group name**	**Group description**	**Dose (per mouse in 100 μl)**
Naïve	Untreated mice	–
PMG5	PMG5 only	1.6 mg
CpG	Soluble CpG B (1826)	50 μg
Ad5-TRP2/PMG5	Ad5-TRP2 with PMG5	1 × 10^8^ PFUs with 1.6 mg of PMG5
Ad5-TRP2/CpG	Ad5-TRP2 with soluble CpG B	1 × 10^8^ PFUs with 50 μg of CpG
Ad5-TRP2/PMG3	Ad5-TRP2 with PMG3	1 × 10^8^ PFUs with 1.6 mg of PMG3
Ad5-TRP2/PMG4	Ad5-TRP2 with PMG4	1 × 10^8^ PFUs with 1.6 mg of PMG4
Ad5-TRP2/PMG5	Ad5-TRP2 with PMG5	1 × 10^8^ PFUs with 1.6 mg of PMG5
Ad5-TRP2/PMG3 ICM	Ad5-TRP2 with PMG3 and αPD-1 and α4-1BB	1 × 10^8^ PFUs with 1.6 mg of PMG3 with 100 μg of αPD-1 and 100 μg of α4-1BB
Ad5-TRP2/PMG4 ICM	Ad5-TRP2 with PMG4 and αPD-1 and α4-1BB	1 × 10^8^ PFUs with 1.6 mg of PMG4 with 100 μg of αPD-1 and 100 μg of α4-1BB
Ad5-TRP2/PMG5 ICM	Ad5-TRP2 with PMG5 and αPD-1 and α4-1BB	1 × 10^8^ PFUs with 1.6 mg of PMG5 with 100 μg of αPD-1 and 100 μg of α4-1BB
αNK Ad5-TRP2/PMG5	Ad5-TRP2 with PMG5 and αNK antibodies	1 × 10^8^ PFUs with 1.6 mg of PMG5 with 150 μg of αNK
αCD4 Ad5-TRP2/PMG5	Ad5-TRP2 with PMG5 and αCD4 antibodies	1 × 10^8^ PFUs with 1.6 mg of PMG5 with 150 μg of αCD4
αCD8 Ad5-TRP2/PMG5	Ad5-TRP2 with PMG5 and αCD8 antibodies	1 × 10^8^ PFUs with 1.6 mg of PMG5 with 150 μg of αCD8
αCD4 αCD8 Ad5-TRP2/PMG5	Ad5-TRP2 with PMG5 and αCD8 and αCD4 antibodies	1 × 10^8^ PFUs with 1.6 mg of PMG5 with 150 μg of αCD8 and αCD4
αNK Ad5-TRP2/PMG5 ICM	Ad5-TRP2 with PMG5 and αPD-1, α4-1BB, and αNK antibodies	1 × 10^8^ PFUs with 1.6 mg of PMG5 with 100 μg of αPD-1, 100 μg of α4-1BB, and 150 μg of αNK
αCD4 Ad5-TRP2/PMG5 ICM	Ad5-TRP2 with PMG5 and αPD-1, α4-1BB, and αCD4 antibodies	1 × 10^8^ PFUs with 1.6 mg of PMG5 with 100 μg of αPD-1, 100 μg of α4-1BB, and 150 μg of αCD4
αCD8 Ad5-TRP2/PMG5 ICM	Ad5-TRP2 with PMG5 and αPD-1, α4-1BB, and αCD8 antibodies	1 × 10^8^ PFUs with 1.6 mg of PMG5 with 100 μg of αPD-1, 100 μg of α4-1BB, and 150 μg of αCD8
αCD4 αCD8 Ad5-TRP2/PMG5 ICM	Ad5-TRP2 with PMG5 and αPD-1, α4-1BB, and αCD8 antibodies	1 × 10^8^ PFUs with 1.6 mg of PMG5 with 100 μg of αPD-1, 100 μg of α4-1BB, and 150 μg of αCD8 and αCD4

Throughout the study, mice were monitored for general signs of distress/toxicity including a reduction in activity/responsiveness to touch, hunched back, glassy coating over the eyes, and significant (≥20%) weight reduction.

### Ex vivo staining of TRP2-specific CD8^+^ T lymphocytes

At 15 days PTC, 180 μl of blood was collected by submandibular bleeding and mixed with ACK buffer (150 mM NH_4_Cl, 10 mM KHCO_3_, and 0.1 mM Na_2_EDTA) to lyse red blood cells. After a 10-min incubation at room temperature, cells were washed (centrifuged at 230*g* twice) using complete growth media. The cells were resuspended at <10^7^/ml [in ice-cold PBS containing 2% (v/v) FCS and 0.05% (w/v) sodium azide:FACS buffer] and transferred to 96-well V-bottomed trays. Cells were then centrifuged at 4°C at 230*g* for 5 min, and then the supernatant was removed. Cell pellets were then resuspended in 50 μl of anti-mouse CD16/CD32 and incubated on ice for 15 min. Then, 50 μl of tetramer stain (MBL, Sunnyvale, CA) diluted 1/100 in FACS buffer was added, and cells were incubated on ice in the dark for 30 min (*21*). Next, 100 μl of antibody cocktail [anti–CD8a-FITC (1/400, clone 53-6.7, eBioscience) and anti–CD3-PECy5 (1/200, clone 145-2C11, eBioscience)] was added to the cells, and cells were then incubated for 20 min on ice in the dark. Cells were then washed twice with FACS buffer and resuspended in 100 μl of Cytofix solution and incubated on ice in the dark for 10 min. Last, 100 μl of 1× Perm/Wash solution (BD Biosciences) was added to each well, then cells were spun at 660*g*, resuspended in FACS buffer, and stored sealed at 4°C until they were acquired using the FACScan flow cytometer and analyzed using FlowJo software.

### Depletion studies

Mice were randomly divided into 10 groups (five mice per group), as described in Table 1. Mice were then challenged on the dorsal right flank with 2 × 10^5^ live B16.F10 cells in 100 μl of serum-free DMEM complete media. The following day, mice designated to receive Ad5-TRP2 were given a single dose contralaterally at 1 × 10^8^ PFUs. On days 8, 11, and 13 PTC, mice were given their designated PM treatments under anesthesia at the site of tumor inoculation on the right dorsal flank of the mouse. Tumor volumes and mice weights were recorded every 2 to 3 days. On days 8, 11, 13, 16, and 18 PTC, select groups were administered with αPD1 (100 μg per mouse per administration) and α4-1BB (intraperitoneally at 100 μg per mouse per administration). On days 6, 7, and 8 PTC then every 3 days until day 30 PTC, designated groups received 150 μg of antibodies (αNK, clone number PK136; αCD4, clone number GK 1.5; αCD8, clone number 2.43) intraperitoneally to deplete specified immune cell populations. Endpoint criteria were met when tumors reached 20 mm in length or width or 10 mm in height. Tumors were assumed to be ellipsoid in shape, and volumes were recorded by measuring the length, width, and height with calipers and calculated using the formula

$$\text{Volume}=(\text{length}\times \text{width}\times \text{height})\times \frac{\pi }{6}$$

### Tumor- and skin-infiltrating CD8^+^ T cells

In this study, 18 C57BL/6J female mice were randomly divided into six groups (*n* = 3 per treatment): naïve (PBS), Ad5-TRP2, Ad5-TRP2/PMG3, Ad5-TRP2/PMG4, Ad5-TRP2/PMG5, and PMG5. Mice were then challenged on the dorsal right flank (subcutaneously) with 2 × 10^5^ live B16.F10 cells in 100 μl of serum-free DMEM complete media (day 0). The following day, mice designated to receive Ad5-TRP2 were given a single subcutaneous dose contralaterally (left flank) at 1 × 10^8^ PFU per mouse. On days 8, 11, and 13 PTC, mice were given their designated subcutaneous treatment under anesthesia peripheral to the tumor on the right dorsal flank. One day later (i.e., day 14 PTC), mice were euthanized, and skin (at the NP injection site) and tumor tissues were collected, washed with 1× PBS, and fixed in 10% neutral buffered formalin. This was followed by embedding the specimens in paraffin blocks and slicing the paraffin blocks into thin sections. Next, the tissue sections were mounted onto microscope slides and subjected to dehydration and rehydration steps for subsequent staining with the basic nuclear stain (Harris hematoxylin, Leica Biosystems, Buffalo Grove, IL) and acidic stain (eosin, Leica Biosystems), respectively (*73*, *74*). Also, tissue specimens (skin and tumor) were stained with anti-mouse CD8 antibody. Hematoxylin and eosin–stained slides were visualized at 20× under an Olympus BX-61 microscope, while CD8-stained slides were imaged using a PANNORAMIC 1000 digital slide scanner (3DHistech Corporation). Positively stained infiltrating CD8^+^ T cells were enumerated using SlideViewer 2.5 software. Counting was performed in three independent 0.3068-mm^2^ areas per sample (total of 0.92 mm^2^ per sample).

### Statistical analysis

Unless indicated otherwise, statistical differences were analyzed by one-way analysis of variance (ANOVA) followed by a Tukey posttest to compare all pairs of treatments. Where indicated, statistical differences were analyzed by two-way ANOVA followed by a Tukey posttest. Survival curves were analyzed using adjustments for multiple comparisons for the log-rank (Mantel-Cox) test using Dunnett’s method. All statistical tests were performed using GraphPad Prism software (version 9.0.0, La Jolla, CA).

## Notes

View/request a protocol for this paper from *Bio-protocol*.
