# Supplementary material for: Cationic nanoparticles enhance T cell tumor infiltration and antitumor immune responses to a melanoma vaccine
Source: Sci Adv. 2022 Jul 20;8(29):eabk3150. doi: 10.1126/sciadv.abk3150 (PMC9299550; doi:10.1126/sciadv.abk3150)
Supplement: Supplementary file 1 — Figs. S1 to S11 [file sciadv.abk3150_sm.pdf]

Science Advances
AAAS

Supplementary Materials for

Cationic nanoparticles enhance T cell tumor infiltration and antitumor immune responses to a melanoma vaccine

Rasheid Smith *et al.*

Corresponding author: Aliasger K. Salem, aliasger-salem@uiowa.edu

Sci. Adv. **8**, eabk3150 (2022)
DOI: 10.1126/sciadv.abk3150

This PDF file includes:

Figs. S1 to S11

Supplementary Figures

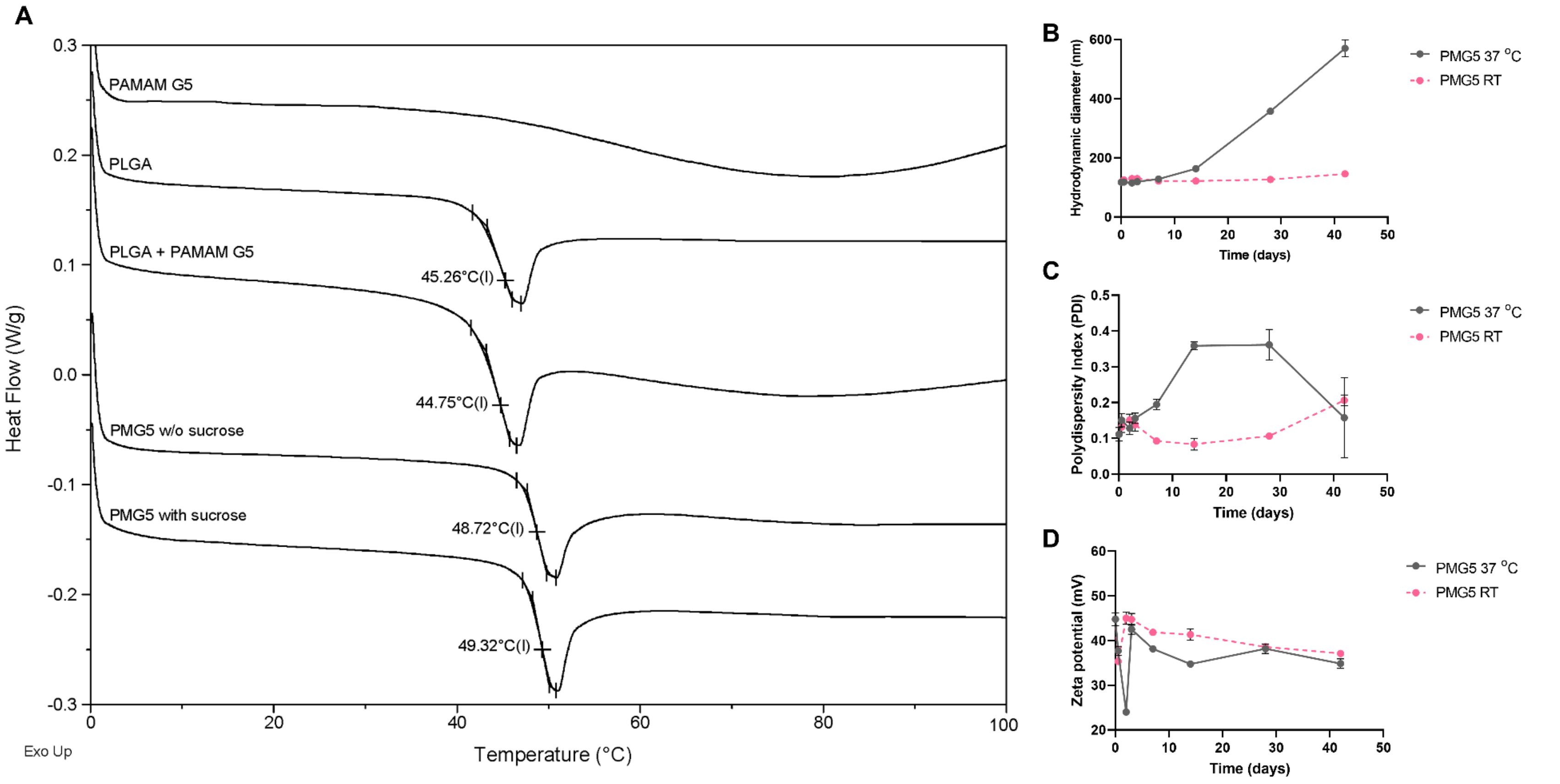

Fig. S1. Thermal Stability of PMG5. (**A**) Differential scanning calorimeter (DSC) thermogram of PMG5 and physical mixture of PLGA and PAMAM. (**B**) Graph showing the size of PMG5 measured overtime at 37 °C and room temperature (RT, 22.5 °C). (**C**) Graph showing the size distribution (polydispersity index) of PMG5 measured over time at 37 °C and RT. (**D**) Graph showing the net surface charge (zeta potential) of PMG5 measured over time at 37 °C and RT. Data are plotted as mean ± SD.

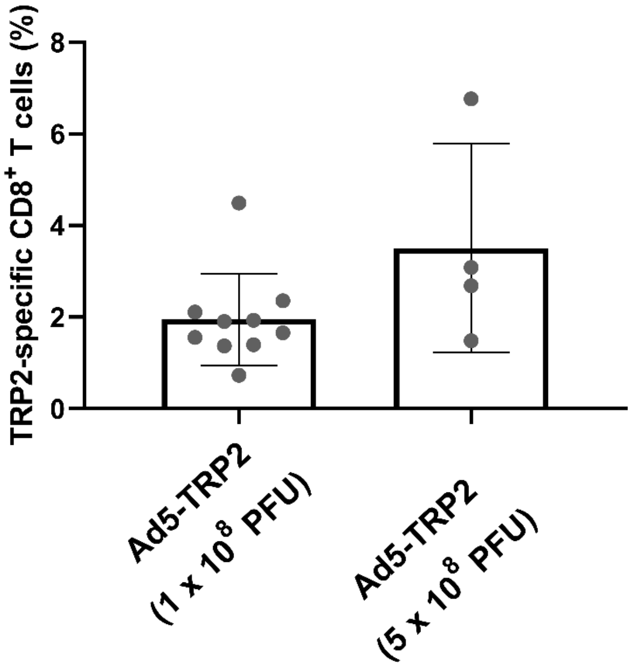

Fig. S2. Levels of TRP2-specific CD8+ T lymphocytes in PBLs from mice following therapeutic vaccination with Ad5-TRP2 at 1 x 10^8 versus 5 x 10^8 PFU. Mice were challenged with B16.F10 cells (day 0) followed by Ad5-TRP2 (1 x 10^8 PFU) or Ad5-TRP2 (5 x 10^8 PFU) vaccination (day 1 PTC). N = 10/group for Ad5-TRP2 (1 x 10^8 PFU) and n = 4/group for Ad5-TRP2 (5 x 10^8 PFU). Levels of TRP2-specific CD8+ T lymphocytes (expressed as a percentage of total CD3+CD8+ T lymphocytes) 14 days post-vaccination in PBLs of mice are shown. Statistical analysis was performed using an unpaired t-test (two-tailed) revealing no significant difference.

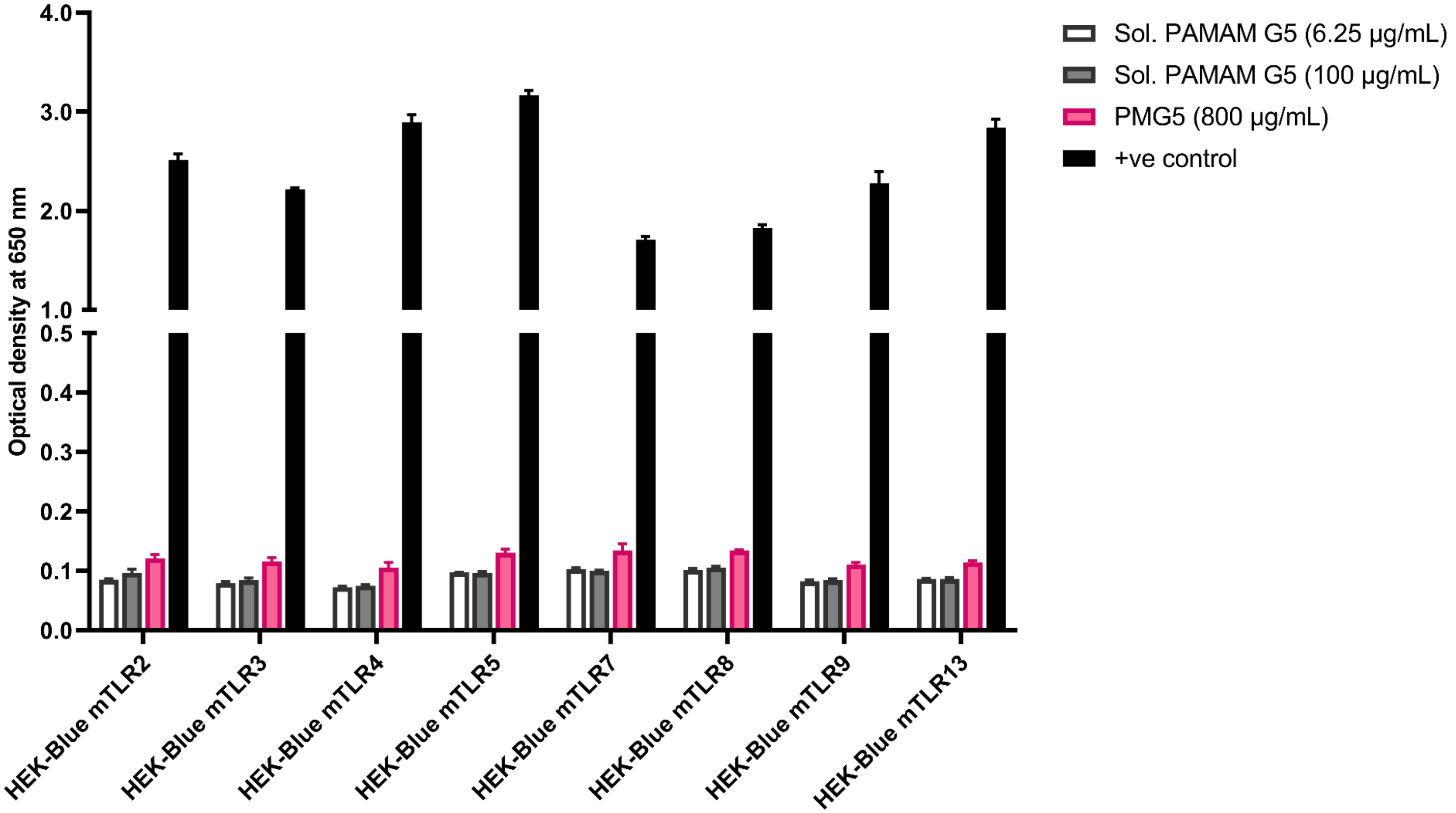

Fig. S3. Mouse TLR agonist screening. PMG5 or soluble PAMAM G5 were incubated at indicated concentrations with TLR-expressing cell lines as described in the *Materials and Methods* section.

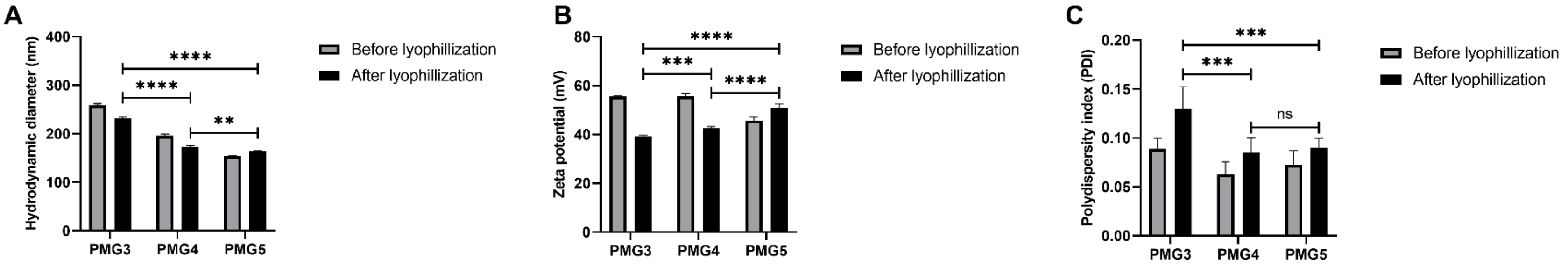

D

	Hydrodynamic diameter (nm)		Zeta potential (mV)		Polydispersity index (PDI)	
	Before lyophilization	After lyophilization	Before lyophilization	After lyophilization	Before lyophilization	After lyophilization
**PMG3**	258.44 ± 3.47	231.68 ± 2.36	55.5 ± 0.36	39.24 ± 0.56	0.089 ± 0.011	0.130 ± 0.022
**PMG4**	195.60 ± 3.84	172.36 ± 3.01	55.64 ± 0.36	42.6 ± 0.64	0.063 ± 0.013	0.085 ± 0.015
**PMG5**	153.58 ± 0.45	163.98 ± 0.61	45.64 ± 1.43	50.96 ± 1.56	0.073 ± 0.015	0.090 ± 0.010

Fig. S4. Characterization of PMG3, PMG4, and PMG5. (**A**) Graph comparing the hydrodynamic diameter of different PM formulations. (**B**) Graph comparing the net surface charge (zeta potential) of different PM formulations. (**C**) Graph comparing the size distribution (polydispersity index) of PM formulations. Data are plotted as mean ± SD. **** = $p < 0.0001$, *** = $p < 0.001$, ** = $p < 0.01$. (**D**) Table comparing the particle characteristics of PMG3, PMG4, and PMG5.

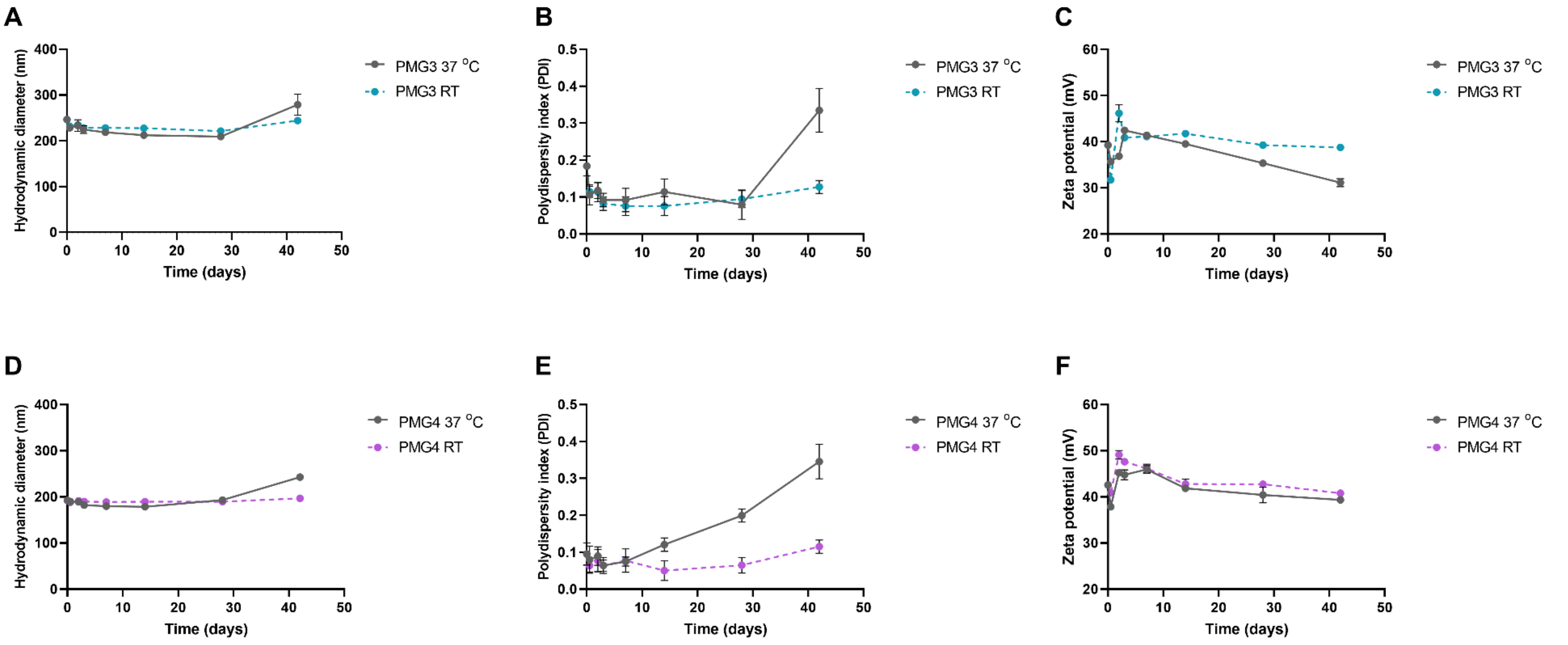

Fig. S5. Stability of PMG3 and PMG4 in Nanopure water at two different temperatures: 37 °C and room temperature (RT, 22.5 °C). (**A**) Graph showing the size measurements of PMG3 over time. (**B**) Graph showing the size distribution measurements (polydispersity index) of PMG3 over time. (**C**) Graph showing the net surface charge (zeta potential) measurements of PMG3 over time. (**D**) Graph showing the size measurements of PMG4 over time. (**E**) Graph showing the size distribution measurements of PMG4 over time. (**F**) Graph showing the net surface charge measurements of PMG4 over time. Data are plotted as mean ± SD.

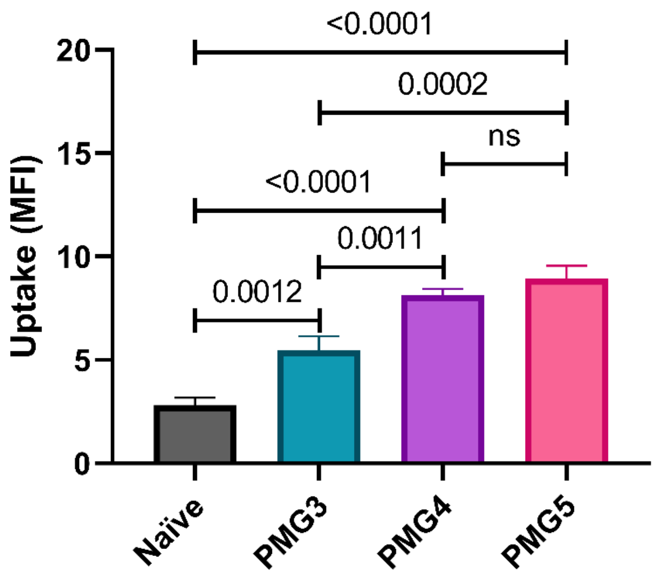

Fig. S6. Graph showing the uptake of PM formulations by BMDCs. BMDCs were incubated with 0.24 mg of different PM formulations for 48 hours. Statistical analysis was performed using one-way ANOVA with Tukey's multiple comparisons test. Error bars represent the standard deviation. The numbers above the graphs refer to the probability as determined by one-way ANOVA with Tukey's post-test. BMDCs were initially seeded in a 12-well plate in media-containing serum for 24 hours and then incubated with PMG3, PMG4, and PMG5 for 48 hours. BMDCs were then collected and analyzed using a FACScan flow cytometer.

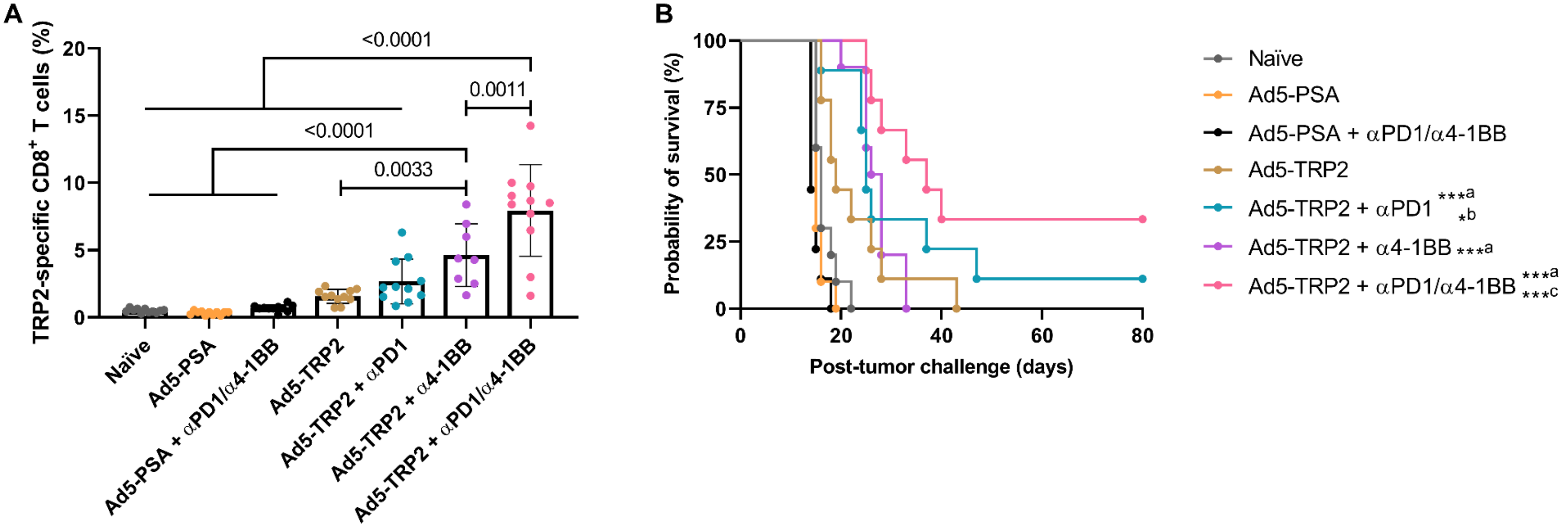

Fig. S7. Levels of TRP2-specific CD8+ T lymphocytes in PBLs following Ad5-TRP2/immune checkpoint therapy. Mice were challenged with B16.F10 cells (day 0) followed by Ad5-TRP2 or Ad5-PSA vaccination (day 1) and then α4-1BB and/or αPD1 (days 8, 10, 13, 16, and 19) (as described in methods). N = 10 per group except where mice were required to be sacrificed due to tumor volume reaching end-point criteria prior to day 14 post-vaccination; or where outliers were removed (**A**) Levels of TRP2-specific CD8+ T lymphocytes 14 days post-vaccination in PBLs of mice treated with indicated combinations. The numbers above the bar refer to the probability as determined by one-way ANOVA with Tukey post-test. (**B**) Survival curve for B16.F10-challenged mice and effect of Ad5-TRP2 and/or immune checkpoint therapy. C57BL/6J mice were challenged with B16.F10 cells (day 0) followed by Ad5-TRP2 or Ad5-PSA vaccination (day 1) and then anti-4-1BB (α4-1BB) and/or anti-PD1 (αPD1) (days 8, 10, 13, 16 and 18) (as described in methods). Statistical significance was determined using the Log-Rank test and then the threshold for *p* was adjusted for multiple comparisons (K = 21). $***^{a}$ = significantly different from naïve and Ad5-PSA groups ($p < 0.002$); $*^{b}$ = significantly different from Ad5-PSA + α4-1BB/PD1 group ($p = 0.033$); $***^{c}$ = significantly different from Ad5-PSA + α4-1BB/PD1 group ($p < 0.002$).

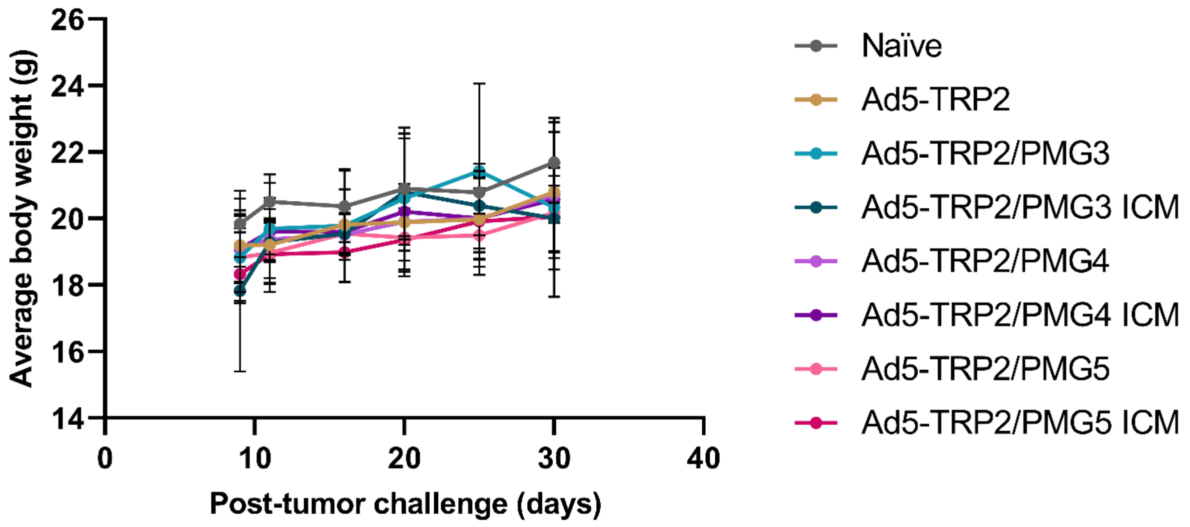

Fig. S8. Graph showing the average weight of mice over time. Body weights were measured on indicated days. The average body weight of mice in the vaccinated groups was similar to the control mice. Data are plotted as mean ± SD.

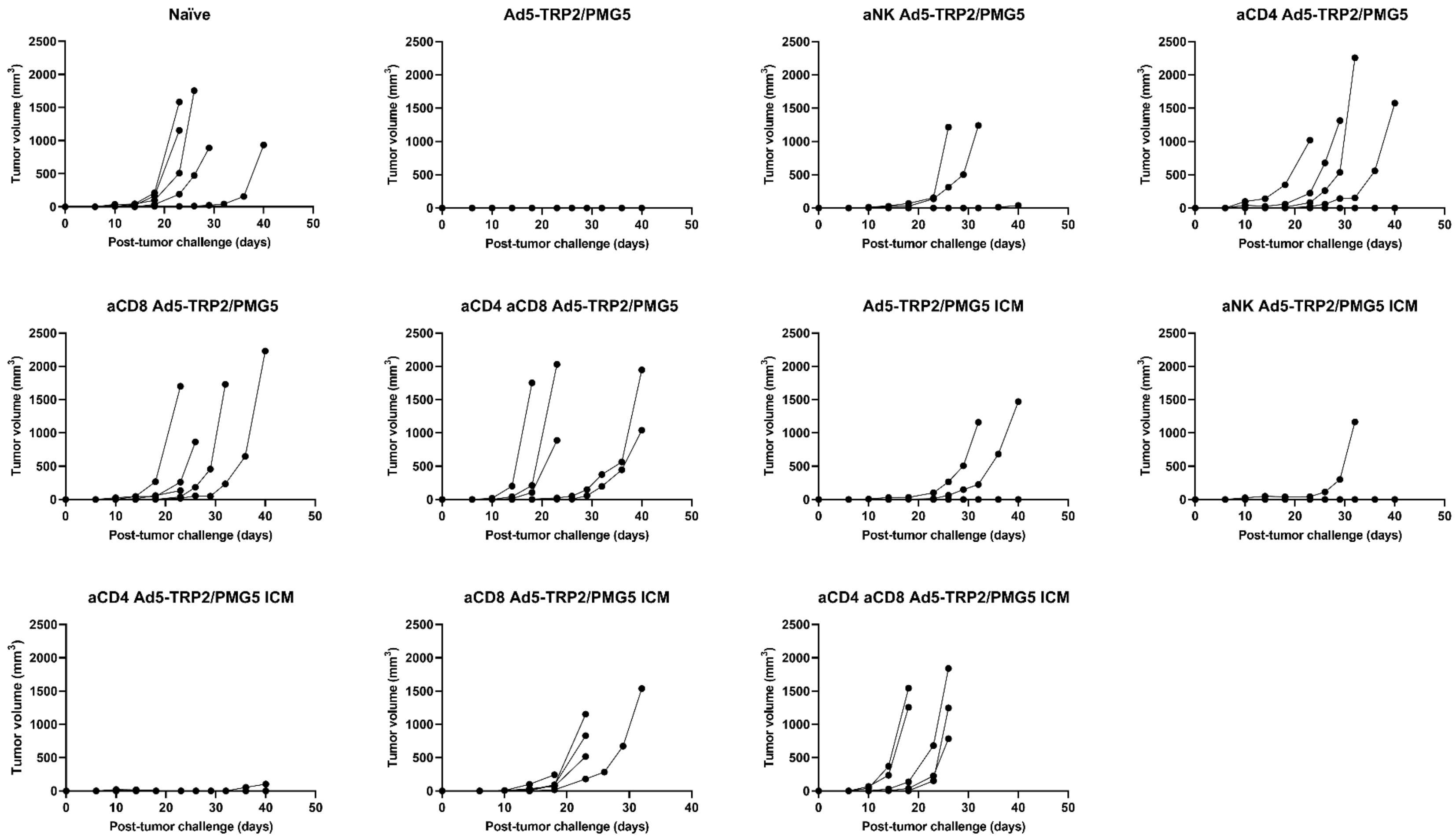

Fig. S9. Evaluation of the effector immune cell population responsible for antitumor efficacy. Tumor volume curves of mice treated with different designated treatments.

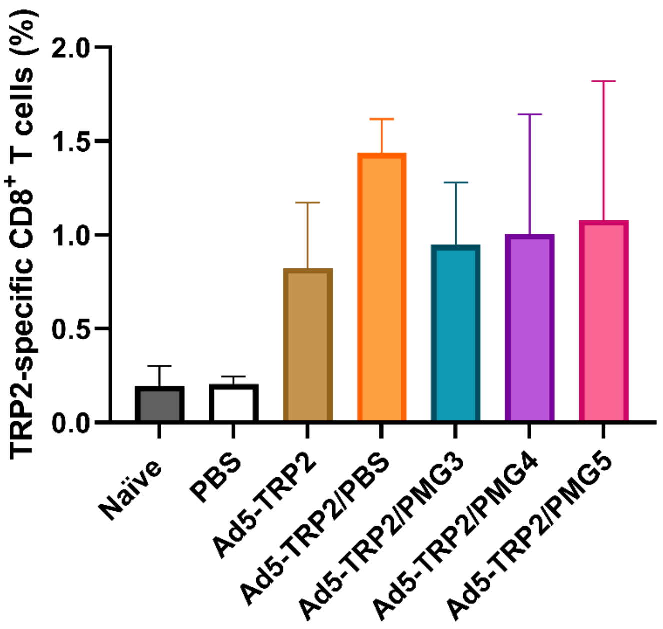

Fig. S10. Graph showing the percent of TRP2+ CD8+ T cells in the peripheral blood (14 days after vaccination in non-tumor bearing mice). C57BL/6J mice (n =3) were vaccinated with Ad5-TRP2 on the left dorsal flank (day 0). On days 7, 9, and 12, mice were given either PBS, PMG3, PMG4, or PMG5 on the right dorsal flank subcutaneously and on day 14 were sub-mandibularly bled and the presence of TRP2-specific CD8+ T lymphocytes evaluated. Data are plotted as mean ± SD.

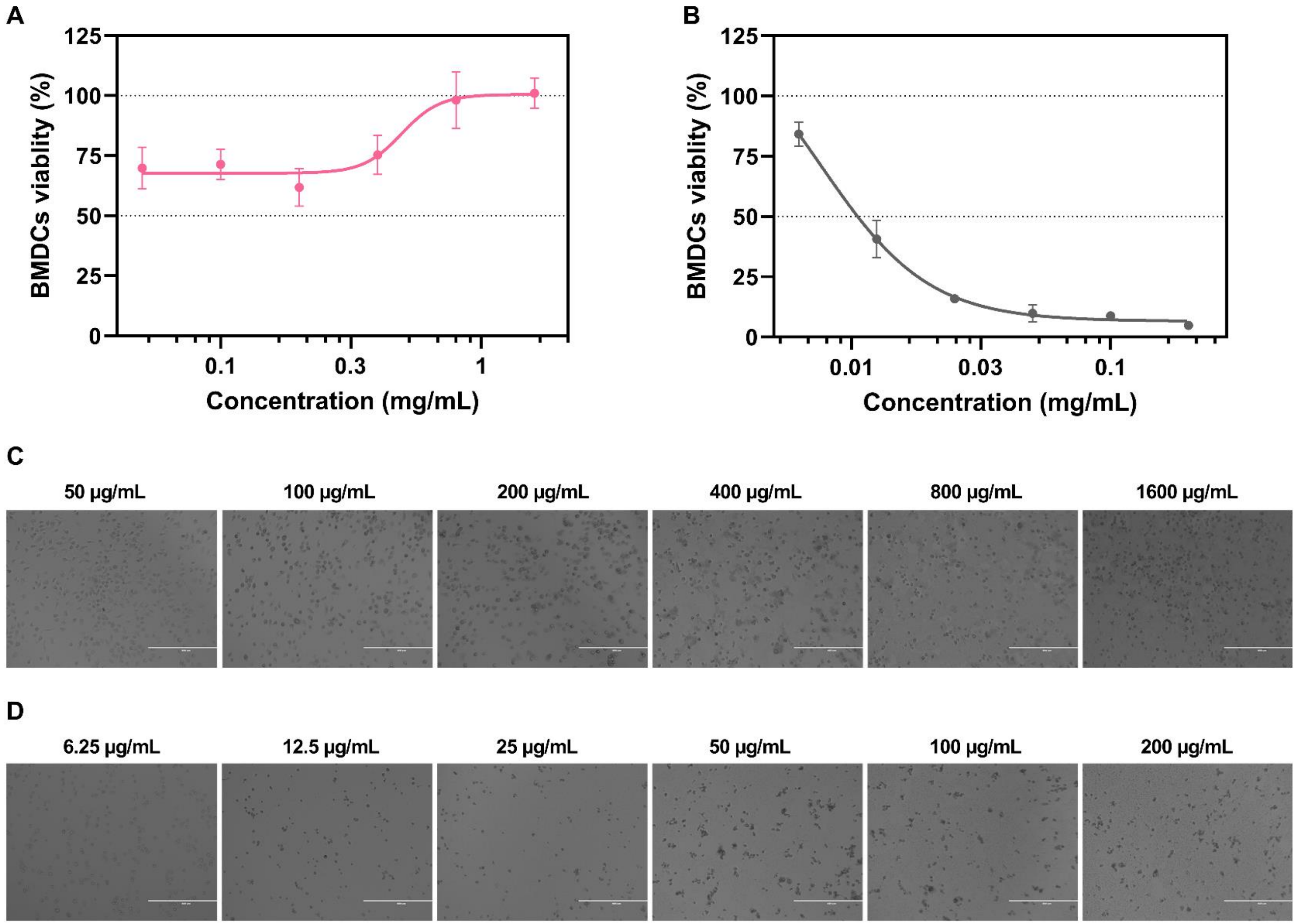

Fig. S11. Cytotoxicity of PMG5 and soluble PAMAM G5 on BMDCs. BMDCs were cultured with (**A**) PMG5 or (**B**) soluble PAMAM G5 (IC_{50} = 7.58 μg/mL) for 48 hours, and then an MTS assay was performed to assess cytotoxicity. (**C**) Images of BMDCs treated with PMG5 at indicated concentrations. (**D**) Images of BMDCs treated with soluble PAMAM G5 at indicated concentrations. Scale bar = 400 μm.
